# Structural basis for cross-group recognition of an influenza virus hemagglutinin antibody that targets postfusion stabilized epitope

**DOI:** 10.1371/journal.ppat.1011554

**Published:** 2023-08-09

**Authors:** Keisuke Tonouchi, Yu Adachi, Tateki Suzuki, Daisuke Kuroda, Ayae Nishiyama, Kohei Yumoto, Haruko Takeyama, Tadaki Suzuki, Takao Hashiguchi, Yoshimasa Takahashi

**Affiliations:** 1 Research Center for Drug and Vaccine Development, National Institute of Infectious Diseases, Shinjuku, Tokyo, Japan; 2 Department of Life Science and Medical Bioscience, Waseda University, Shinjuku, Tokyo, Japan; 3 Laboratory of Medical Virology, Institute for Life and Medical Sciences, Kyoto University, Kyoto, Japan; 4 Laboratory of Precision Immunology, Center for Intractable Diseases and ImmunoGenomics research, National Institutes of Biomedical Innovation, Health and Nutrition; Saito-Asagi, Ibaraki City, Osaka, Japan; 5 Computational Bio Big-Data Open Innovation Laboratory (CBBD-OIL), National Institute of Advanced Industrial Science and Technology, Shinjuku, Tokyo, Japan; 6 Research Organization for Nano and Life Innovation, Waseda University, Shinjuku, Tokyo, Japan; 7 Institute for Advanced Research of Biosystem Dynamics, Waseda Research Institute for Science and Engineering, Waseda University, Shinjuku, Tokyo, Japan; 8 Department of Pathology, National Institute of Infectious Diseases, Shinjuku, Tokyo, Japan; Icahn School of Medicine at Mount Sinai, UNITED STATES

## Abstract

Plasticity of influenza virus hemagglutinin (HA) conformation increases an opportunity to generate conserved non-native epitopes with unknown functionality. Here, we have performed an in-depth analysis of human monoclonal antibodies against a stem-helix region that is occluded in native prefusion yet exposed in postfusion HA. A stem-helix antibody, LAH31, provided IgG Fc-dependent cross-group protection by targeting a stem-helix kinked loop epitope, with a unique structure emerging in the postfusion state. The structural analysis and molecular modeling revealed key contact sites responsible for the epitope specificity and cross-group breadth that relies on somatically mutated light chain. LAH31 was inaccessible to the native prefusion HA expressed on cell surface; however, it bound to the HA structure present on infected cells with functional linkage to the Fc-mediated clearance. Our study uncovers a novel non-native epitope that emerges in the postfusion HA state, highlighting the utility of this epitope for a broadly protective antigen design.

## Author summary

Induction of broadly protective antibodies is a clue to combat the antigenic variation of influenza virus. In this context, directing the antibody specificity toward the conserved hemagglutinin (HA) epitopes is a rational approach; however, it is hampered by virus strategies to hide the vulnerable sites. Here, we show a novel non-native epitope that emerges in the postfusion HA state with unique structure, denoted as kinked loop-helix. Antibody toward this epitope is accessible to HA on infected cells, demonstrating an exposure of the vulnerable sites during viral replication. In-depth structural analysis uncovers how antibody adapts to the conserved non-native epitope and provide cross-group protection, with an implication to rational design of broadly protective immunogens.

## Introduction

Influenza A virus (IAV) is one of the antigenically evolving pathogens able to cause infection in humans, leading to seasonal epidemics and sporadic cross-species transmission [1]. The most common variation in IAV strains occurs in a surface glycoprotein, hemagglutinin (HA), which serves as the major target of protective antibodies. Based on its sequence, HA can be classified in 18 subtypes, which are further subdivided into two phylogenetic groups, 1 and 2, with group 1 represented by the seasonal H1 and zoonotic H5 subtypes and group 2 represented by the seasonal H3 and zoonotic H7 subtypes [2–4]. Most antibodies elicited by vaccination or infection target the epitopes in immunogenic head domain. However, the elicitation of head-targeting antibodies often exhibits limited breadth of protection, owing to the hyper-variable nature of head domain [5].

Despite the ability of IAV to evade antibodies, the HA preserves conserved regions among multiple subtypes, and antibodies targeting these sites offer a broader cross-protection against diverse viral strains [6]. The discovery of broadly reactive antibodies in the past decade allowed for the identification of two classes of conserved neutralizing epitopes, receptor binding site (RBS) epitopes in the head [7] and conformational stem (CS) epitopes in the stem [8]. These epitopes, especially CS, confer heterosubtypic conservation and are present on the conformationally intact HA surface. Therefore, several studies have attempted to stabilize the native HA structure for inducing broadly reactive antibodies targeting these conserved epitopes [9, 10].

Recent reports have uncovered two additional classes of non-neutralizing, yet highly conserved, sites within the native HA trimer, the head interface (HI) [11–13] and the long alpha helix (LAH) epitopes [14], inside the head and stem, respectively. Based on structural analysis of antibody-epitope complex, the presentation of HI epitopes is speculated to occur upon subunit dissociation like “breathing” [15]. On the other hand, HA split vaccines modulated by acidic condition increased the accessibility by LAH monoclonal antibodies (mAbs) [14], showing that LAH presentation is promoted by conformational changes leading to the conversion to a non-native, postfusion state [16, 17]. Although human LAH mAbs exhibit intra-group 2 reactivity and heterosubtypic protection in an IgG Fc-dependent manner [14], several questions remain unanswered whether its cross-protection properties can be extended to both HA subgroups, and how LAH antibodies target the non-native HA epitopes and provide protection.

In this study, we characterized a novel human LAH mAb, named LAH31, from a panel of human mAbs obtained in a previous study [14]. Contrary to previously described LAH mAbs, LAH31 exhibited a broad cross-group recognition against seasonal H1 and H3 subtypes. Crystallographic analysis revealed that the LAH31 epitope enclosed in a narrow region of LAH, which we named the kinked loop-helix (KLH) region. This region is fully exposed and stabilized in the postfusion state. Despite the occluded nature of the epitope in the native prefusion HA, LAH31 confers robust cross-group protection, not through neutralization but via Fc-dependent mechanisms, mediated by the recognition of this non-native epitope likely presented on infected cells. The LAH31 binding site is well conserved among all HA subtypes in group 2, and in approximately half the subtypes in group 1. Furthermore, our in silico docking analysis highlighted the distinct contribution of hydrogen bonds to the binding specificity of LAH mAb and this epitope. The structural features of non-native HA epitopes for cross-group protection will provide new insights for the development of a next-generation vaccine to confer universal protection against IAV.

## Results

### Human HA stem helix antibody, LAH31, shows extraordinary cross-group reactivity and confers protection in an Fc-dependent manner

**Fig 1:**
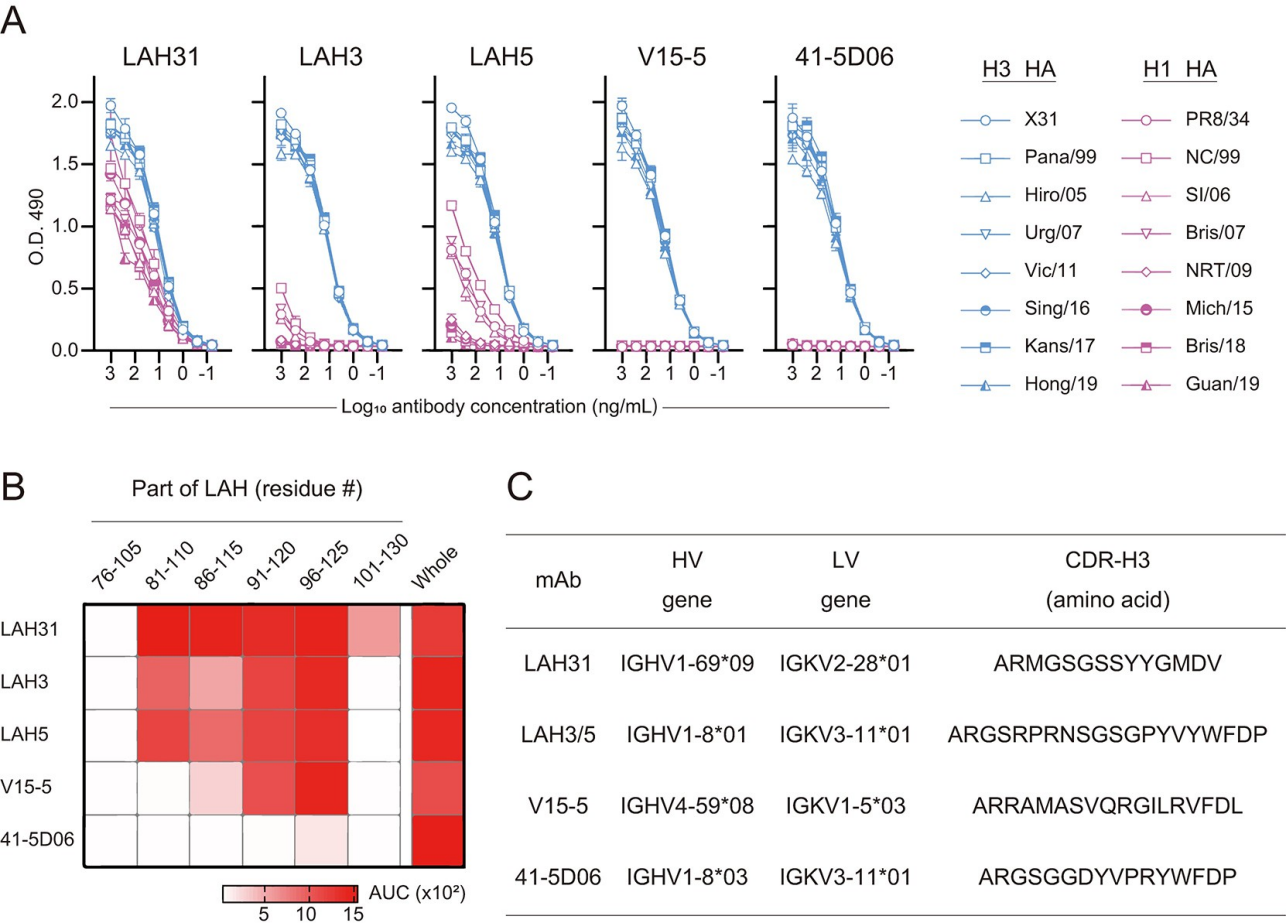
Identification of LAH31 as a novel cross-group protective LAH mAb. (A) LAH mAbs were tested for binding activity against H1 (pink) and H3 (blue) subtypes using ELISA. Full strain names of individual HA are detailed in S1 Table. LAH3, LAH5, V15-5, and 41-5D06 are published LAH clones. The assay was conducted in duplicates and the representative data from two independent experiments are represented as mean ± SD. (B) The binding region of LAH mAbs was investigated using ELISA with the 30-residue overlapping LAH peptides derived from A/Hong Kong/1/68 (H3N2). The amino acid number of the corresponding region is defined above each column in H3 numbering. The binding activity against the whole LAH peptide (amino acids: 76–130) was also assessed. Binding of individual mAb to each antigen is color-coded according to the AUC of the antibody dilution curve as indicated in the color legend. The assay was conducted in duplicates and representative data from two independent experiments are represented. (C) Variable gene usage of V segments in heavy and light chains and CDR-H3 sequence of the individual LAH mAbs. CDR region is defined by IMGT.

We have selected a human mAb panel with specificity to LAH region of HA2, including four previously characterized mAbs, LAH3, LAH5, V15-5, and 41-5D06 [14, 18, 19], and the newly characterized mAb, LAH31. The binding breadth of the antibodies to multiple HA antigens from H3N2 and H1N1 isolates was assessed using direct ELISA (Fig 1A). The mAbs used in this study were originally isolated based on H3 HA binding with the homosubtypic breadth [14]. The binding breadth of these LAH mAbs covered eight H3 HA proteins from H3N2 strains, isolated from 1968 to 2019 seasons. Three LAH mAbs (LAH3, LAH5, and LAH31) exhibited cross-reactivity to H1 HAs in group 1, LAH31 showing the most prominent binding breadth. The core epitope targeted by the LAH mAbs partially overlapped (Fig 1B). We located the LAH31 core epitope to the HA 101–110 residues, based on the binding data. LAH3 and LAH5 shared the clonotype, with the same CDR3 and V_H_/V_L_ usage (Fig 1C). In contrast, LAH31 showed a unique clonotype encoded by IGHV1-69 gene, a common gene in broadly neutralizing antibodies against diverse types of viral antigens, including SARS-CoV-2 spike, HIV Env, and influenza virus HA [20, 21]. Recently, IGHV1-69 alleles using leucine in their CDR-H2 loops, represented by IGHV1-69*09, have been shown to bear substantial polyreactivity or autoreactivity [22]. We observed that the levels of polyreactivity were comparable to those of HA stem antibody clone, FI6, which has detectable autoreactivity but limited polyreactivity to other antigens [23]; however, the autoreactivity/polyreactivity was much weaker than that of a reference polyreactive antibody [24] (S1 Fig).

**Fig 2:**
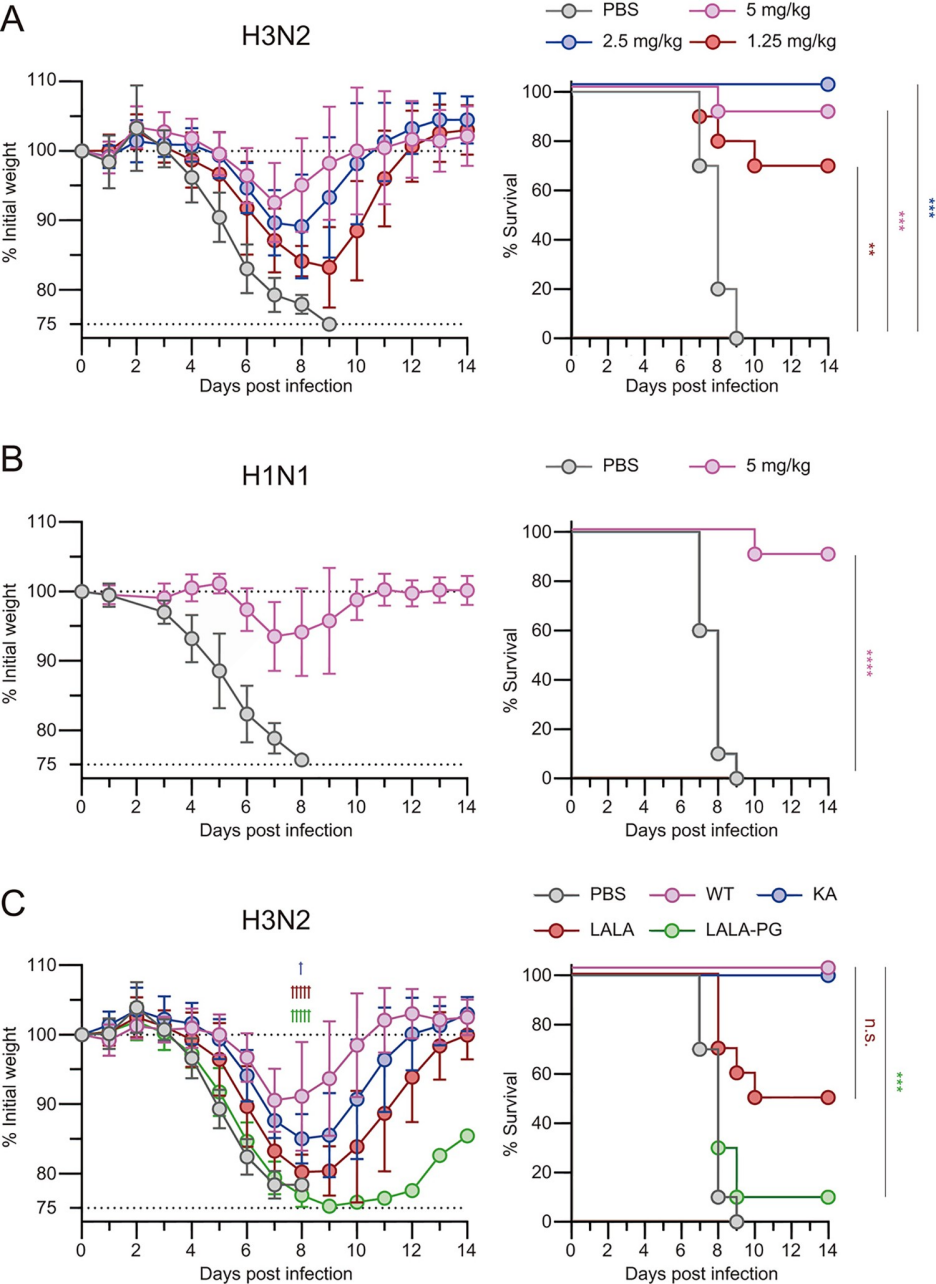
Cross-group protection provided by LAH31. (A-C) C57BL/6 mice were treated with (A and B) indicated dose of LAH31 IgG2c or PBS or (C) 5 mg/kg of LAH31 Fc variants (IgG2c-WT, -KA, -LALA, or LALA-PG) or PBS. After 3 h of antibody injection, mice were infected with (A and C) X31 (H3N2) or (B) A/Guangdong-Maonan/SWL1536/2019 (H1N1) viruses. Mice presenting a body weight loss higher than 25% of the initial weight were euthanized. The combined data from two independent experiments (n = 10 for each group) are represented. Each plot indicates the data at the indicated time points. Values represent the mean ± SD. The *P* values were determined using a two-way ANOVA (body weight loss) or log-rank (survival curve) tests. For multi-group comparisons of survival curves, statistical significance was adjusted by Bonferroni correction. (A) **p < 0.0017 and ***p < 0.00017, compared with the PBS group. (B) ****p < 0.0001, compared with the PBS group. (C) †*p* < 0.05 and ††††*p* < 0.0001 for weight loss; ***p < 0.0001 for survival, LAH31 IgG2c-WT compared with LAH31 IgG2c-KA, -LALA or -LALA-PG.

To determine whether LAH31 could protect against influenza virus infection, we carried out an in vivo protection study in mice, using lethal virus challenges. Mice infused with LAH31 (mouse IgG2c subclass) circumvented the body weight loss in dose-dependent manner and exhibited higher survival rates up to 2 weeks after infection with the H3N2 (X31) virus (Fig 2A). The infused mice were then challenged with lethal dosage of the group 1 H1N1 (Guangdong-Maonan) virus (Fig 2B). The infused LAH31 provided the protection against both viral strains, demonstrating the cross-group protective properties.

LAH31 exhibited no neutralizing activity against the H3N2 strain in a micro-neutralization assay (S2 Fig), consistent with earlier finding from other LAH antibodies [14]. To gain mechanistic insights on the LAH31-mediated protection in vivo, we created LAH31 IgG2c mutants showing: an attenuated binding to the complement (KA mutation) [25]; attenuated ability to activate the FcγR and partially the complement (LALA mutations) [26]; and attenuated ability to activate FcγR plus complement (LALA-PG mutations) [27]. The mutated LAH31 IgG antibodies equally bound to plate-bound HA proteins, excluding the indirect effects on binding resulting from Fc mutations (S3 Fig). Mice infused with 5 mg/kg of individual LAH31 IgG mutants were challenged with lethal H3N2 dose and the protective effect of the antibody was evaluated through morbidity (body weight loss) and mortality (survival rate) (Fig 2C). The LAH31-infused mice circumvented the morbidity at differential levels in an Fc-mutation dependent manner. The body weight loss was more severe in the LALA-PG group, and lower in the KA group (intermediate in the LALA group). Body weight loss was correlated with the survival rates in the two weeks after the viral infection. The subtle protective function of the LAH31 KA mutant on morbidity suggested the mechanistic involvement of complement; however, the reduced survival rate in LALA and even more pronounced in LALA-PG mutants demonstrated the major contribution of activating FcγR and a complementary role of the complement in this protection mechanism.

### LAH31 targets the kinked loop-helix epitope which is presented after conversion to the postfusion state upon infection

**Fig 3:**
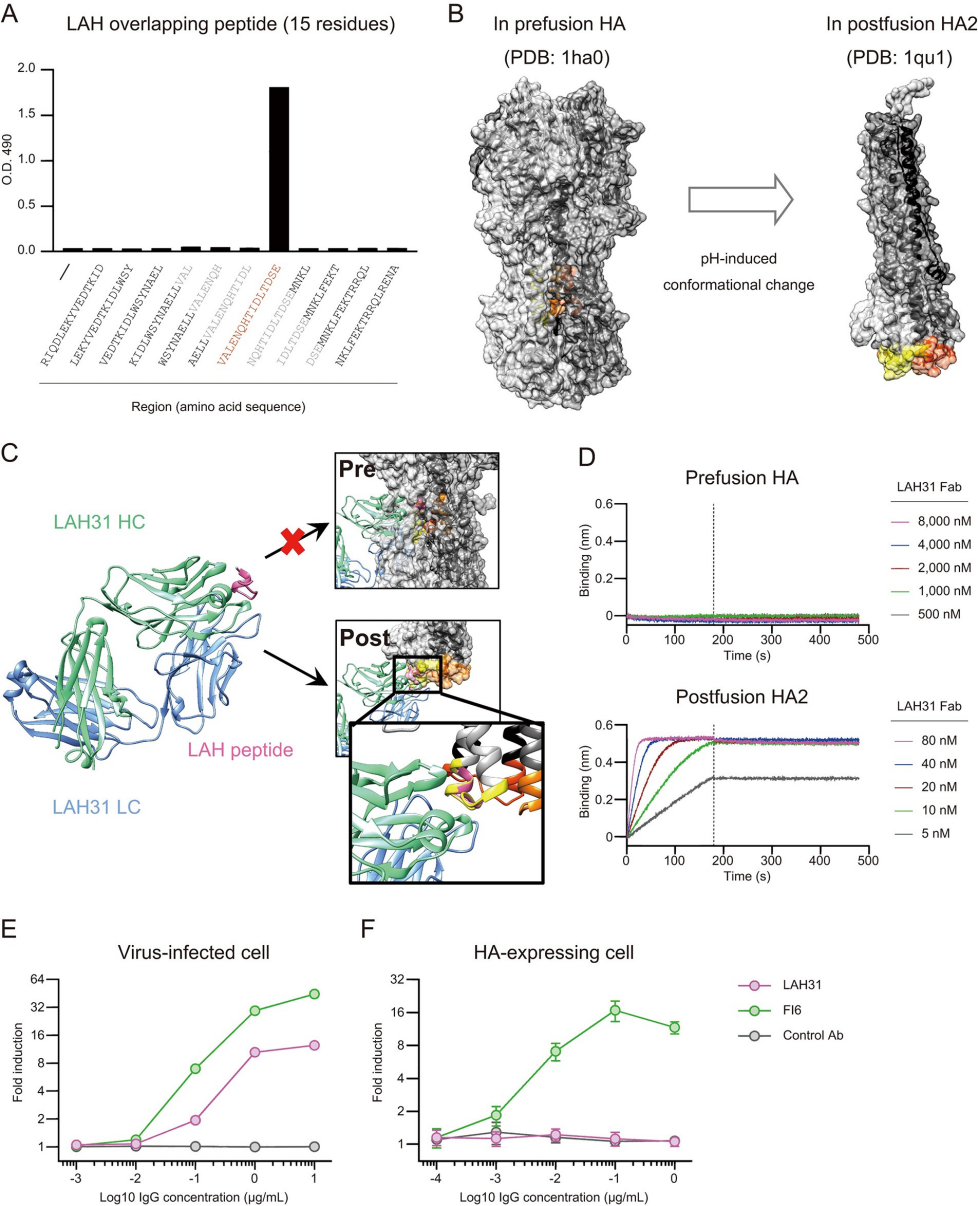
LAH31 epitope is concealed in prefusion state but exposed and stabilized upon the postfusion conversion. (A) Binding region of LAH31 was investigated using ELISA with 15-residue overlapping LAH peptides. Amino acid sequence of individual peptides is indicated below each bar. Sequence corresponding to LAH31 binding is colored in red. Peptides sharing core sequences, yet undetectable by LAH31, are partially colored in white. The assay was conducted in duplicates and representative data from two independent experiments are represented as mean ± SD. (B) The core region of LAH31 epitope is highlighted in the schematic model of the trimeric form of the prefusion HA (PDB: 1ha0) or postfusion HA2 (PDB: 1qu1). Each HA protomer or LAH31 epitope region are colored in black, grey, and white or in yellow-red, orange, and yellow, respectively. (C) The crystal structure of LAH31 Fab complexed with LAH epitope is shown in the left (green, LAH31 heavy chain; blue, LAH31 light chain; pink, epitope LAH peptide). The sequence of the corresponding peptide is the same as (A). Superimposition of LAH31 complex on the prefusion HA or postfusion HA2 illustrated in (B) is shown in the right. (D) LAH31 Fab binding activity against the native prefusion HA or postfusion HA2, measured using biolayer interferometry. (E and F) In vitro ADCC activity of LAH31. (E) X31 virus-infected MDCK cells or (F) HA-expressing EL4 cells were incubated with serially diluted mAbs (LAH31, FI6, irrelevant control antibody) in the presence of mouse FcγRIV-expressing reporter cells. The assay was conducted in duplicates and representative data from two independent experiments are represented. Values represent mean ± SD fold induction of luminescence relative to the no IgG control.

We conducted epitope analysis using 11 overlapping peptides with 15 amino acid length to narrow the core epitope region of LAH31 (Fig 3A). LAH specifically bound to one peptide (100–114 residue) and failed to bind the others despite the overlapping 10 amino acids. The location of the LAH epitope under the prefusion and postfusion HA conformation suggests that the epitope is only exposed in the postfusion HA2 (Fig 3B). We located the LAH31 epitope to the bottom tip of the postfusion HA2, which obtains a unique conformation with the KLH structure resulting from postfusion conversion. To gain further insight on the structural events of epitope recognition, we determined the X-ray crystal structure of the LAH31 Fab bound to the epitope peptide, at the resolution of 1.95 Å (Fig 3C and S2 Table). The binding structure supports the inaccessibility of LAH31 to the epitope in the native prefusion HA conformation; however, this epitope becomes accessible in the postfusion HA2. The architecture of the epitope peptide in the LAH31-bound complex matched with the KLH region observed in the crystal structure of the postfusion HA2 [28]. Therefore, the LAH31 epitope can be defined as a new class of non-native epitopes, arising after postfusion conversion.

To further confirm the binding specificity of LAH31 to the postfusion HA, we determined the binding to the prefusion stabilized HA and the postfusion HA2 attached to a sensor through biotin-streptavidin (SA). Of note, the postfusion HA2 antigen was designed by extracting whole HA1 and fusion peptide of HA2, enabling the expression of soluble HA2 fragment in the postfusion state without acidic treatment [28] (S3 Table). LAH31 Fab failed to bind to the prefusion stabilized HA above the detection level, but bound to the sensor coated with postfusion HA2 (Fig 3D). In line with this, we observed little binding of LAH31 against commercial influenza split vaccine, which would be dominated with prefusion form of viral HAs (S4 Fig). Together, these data demonstrate that LAH31 recognizes the epitope only under the postfusion stabilized conformation, not in the native prefusion, owing to the occlusion of the epitope in this conformation.

Considering that the LAH31 epitope does show KLH structure solely after the postfusion conversion, it is important to assess the conditions for antibody accessibility of the non-native epitope and consequent protection. Therefore, the antibody accessibility was evaluated based on ADCC activity toward the HA antigens on two types of cell lines; MDCK cell lines infected by H3N2 viruses with trypsin treatment and EL4 cell lines expressing H3 trimeric HA without trypsin treatment. FI6, which binds to the stem epitope exposed in prefusion HA [8], showed substantial ADCC activity on virus-infected cells and the transfected cells (Fig 3E and 3F), indicating that FI6 stem epitope was presented in the proper HA conformation in both infected and transfected cells. In contrast, the HA on infected cells, but not on transfected cells, induced ADCC activity of LAH31, suggesting that the LAH31 epitope was likely presented in the HA of infected cells in the way to activate ADCC pathway. Therefore, these data show that virus-infected cells can present the non-native LAH31 epitope and allow the cell marking by LAH31, which could promote the IgG-mediated effector function against infected cells in vivo (Fig 2C).

### Structural analysis of LAH31-epitope complex accounts for the cross-group HA reactivity

**Fig 4:**
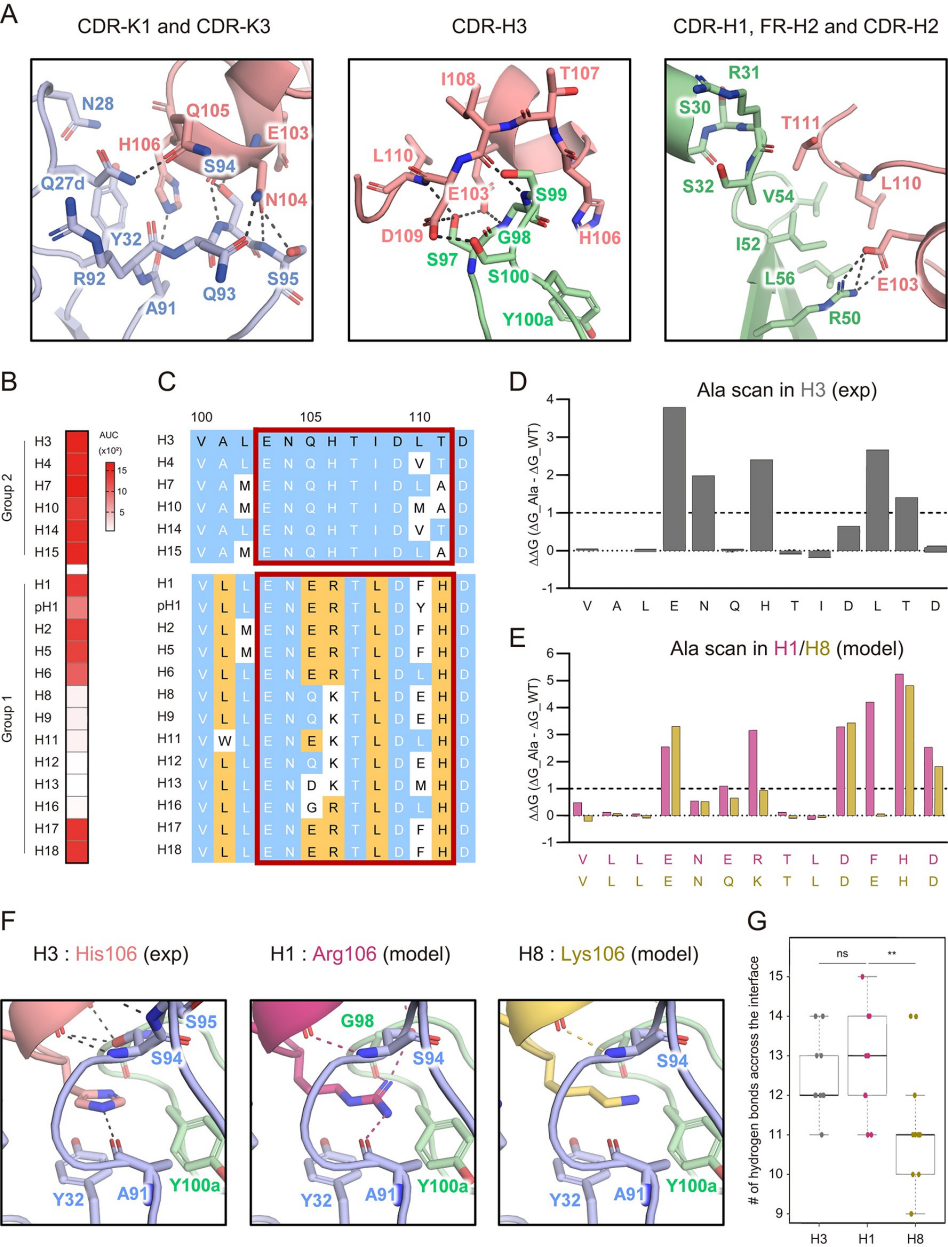
Crystallographic and computational analyses reveal the key foot prints for the cross-group recognition of LAH31. (A) Detailed recognition mode of the CDR-K1 and CDR-K3 (left), CDR-H3 (middle) and CDR-H1, and FR-H2 and CDR-H2 (right) by LAH31. Contact residues are defined by location within 4 Å between LAH31 and epitope peptide (S4 Table). Polar interactions are indicated by dot lines. Residues are colored as Fig 3 and numbered in Kabat and H3 numbering for LAH31 and the peptide, respectively. (B) Binding breadth of LAH31 was tested through ELISA using 18 HA subtypes, including both pre- and post-pandemic strains for H1. Full strain names are detailed in S1 Table. Binding of LAH31 is color-coded as Fig 1B. (C) Amino acid sequence alignment of the epitope region in the HAs tested in (B). Residues same as H3 sequence are colored in white and highlighted in blue, and those different from H3 but conserved in both H1 strains are highlighted in yellow. Contact residues on H3 and corresponding sequences of other subtypes are boxed in red. The sequence is numbered above in H3 numbering. (D and E) In silico alanine scanning of the epitope region in H3 (grey) simulated from experimental structure (D) and in H1 (magenta) and H8 (yellow) simulated from model structures (E). Changes in free energy in LAH31 binding were calculated by replacing each residue with Ala and are represented as bar graphs. The amino acid sequences are indicated below each bar. (F) Detailed recognition mode against the 106th residue of the epitope peptide in the H3 experimental structure (left) and in H1 (middle) or H8 (right) model structures. (G) Numbers of hydrogen bonds were calculated from LAH31 complexed with epitope peptides of H3 (grey), H1 (magenta) and H8 (yellow) model structures. Value in the 10 model is plotted. The *P* values were determined using the two-tailed Mann–Whitney U test. Not significant (ns), **p < 0.01. (E-G) Model structures (200) of LAH31-peptide complex were produced in silico and (E and F) the highest model or (G) 10 highest models were chosen for representation.

The structural analysis of the LAH31-epitope complex revealed the contact residues located within 4 Å distance (S4 Table). LAH31 recognized the epitope peptide using CDRs (except CDR-K2) and one FR-H2 residue (Fig 4A). CDR-K1 and CDR-K3 form several hydrogen bonds, such as Gln27d-Gln105, Ala91-His106, Ser94-Glu103, Ser94-Asn104, and Ser95-Asn104. Tyr32 in the CDR-K1 forms a π–π interaction with the His106 of the peptide. CDR-H3 recognizes the short loop of the peptide, from His106 to Asp109, and forms a hydrogen bond network with the main chains of Glu103, Ile108, Asp109, and Leu110 on the peptide. Arg50 of FR-H2, adjacent to CDR-H2, forms a salt bridge with Glu103 of the peptide. The Ile52, Val54, and Leu56 of CDR-H2 form hydrophobic interactions with the Leu110 on the peptide. Finally, CDR-H1 recognizes Thr111 of the peptide via its main chain.

We then investigated LAH31 cross-group reactivity with 18 HA subtypes using ELISA (Fig 4B). We used plates coated with the full-length trimeric HA proteins, because the direct coating permitted LAH31-HA binding, while SA-mediated coating prevented the binding (S5 Fig). Similarly, the binding of S5V2-29 mAb to the trimeric HA coated with SA was attenuated, as this mAb targets an occluded epitope in the HI [12]. Therefore, when the trimeric HA proteins were directly coated into the plates, there was likely conformational change that exposed the occluded epitopes for LAH31 and S5V2-29. Using the direct coated plates, LAH31 bound to all group 2 HA subtypes and a subgroup of group 1 HA subtypes (H1, H2, H5, H6, H17, and H18); however, it failed to bind other subtypes of group 1 (H8, H9, H11, H12, H13, and H16) which form a distinct subcluster based on HA phylogenetic trees (S6 Fig). The cross-group breadth of LAH31 was sufficient to cover all HA subtypes that have caused human diseases in the past (H1, H2, H3, H5, H6, H7, and H10), except for H9 [1]. LAH homologous sequences among all HA subtypes were aligned with the LAH31 contact residues (Fig 4C).

To determine the LAH residues for LAH31 recognition, we conducted in silico alanine scanning to the corresponding peptides from H3, H1, and H8, revealing different interface energies to LAH31 (Figs 4D, 4E and S7). This analysis was based on the H3 crystal structure, and docking calculations for H1 and H8 allowed the construction of these structural models (S7 Fig). The in silico alanine scanning of the H3 peptide-LAH31 mAb complex were also compared between crystal structure and docking models, showing the overall reproducibility of the docking calculation (Figs 4D and S8). While the accuracy of computational prediction remains a matter of debate, the approach utilized in this study represents a state-of-the-art method that is widely employed to address a range of protein design and biological problems [29–33]. The in silico alanine scanning of the H3 peptide-LAH31 complex highlighted the Glu103, Asn104, His106, and Leu110 as the determinants for the recognition of the H3 subtype by LAH31. These residues are involved in hydrogen bonds, π–π interaction, salt bridge, and hydrophobic interactions. In particular, the residues in the positions 106 and 110 accounted for the LAH31 breadth, as those residues are either substituted or variable in the LAH31 non-binding subtypes (Fig 4C). Concordantly, we observed relatively favorable energy in His106 (H3)/Arg106 (H1) and Leu110 (H3)/Phe110 (H1), compared to that in Lys106 (H8) and Glu110 (H8), likely accounting for the LAH31 binding to H3 and H1, but not H8 subtypes (Fig 4D and 4E).

In the H1 subtype, His106 is replaced by Arg106; however, a hydrogen bond is retained in the H1 model structure (Fig 4F). Similar to the His106 of H3 subtype, Arg106 highly contributes to the interaction with LAH31 (Fig 4D and 4E). In contrast, the contribution of the conserved Glu103 and Asn104 residues for binding is lower in H1 than that in H3, and instead, the contribution of the LAH C-terminal region for binding is increased. The comparison of the crystal structures between the H3 and H1 postfusion forms highlighted structural differences at the LAH N-terminal regions (S9 Fig) [28, 34]. In addition, hydrogen bond formation with the residue at the 105th position is also determinant for the binding to group 1 HAs (Fig 4D and 4E). In accordance, LAH31 recognized H6 subtype, but not H16, which exhibits a glycine at the 105th residue (Fig 4B and 4C).

Contrary to the H3 and H1 subtypes, the H8 was not recognized by LAH31. This subtype exhibits a loss of hydrogen bonds between Lys106 and the surrounding amino acids, contributing to lower LAH31 binding (Fig 4F). Overall, the number of hydrogen bonds formed at the interface is significantly reduced for the H8 subtype, compared to the H3 and H1 subtypes (Fig 4G). Furthermore, the hydrophobic residues at the 110th position in H3 (Leu) and H1 (Phe) subtypes showed significant energetic contribution to LAH31 binding, whereas in H8, this position is occupied with a hydrophilic Glu residue (Fig 4E). These differences in amino acids may explain the differences in the specific recognition and binding of LAH31.

### LAH31 acquires cross-group reactivity via somatic hypermutations in the light chain

**Fig 5:**
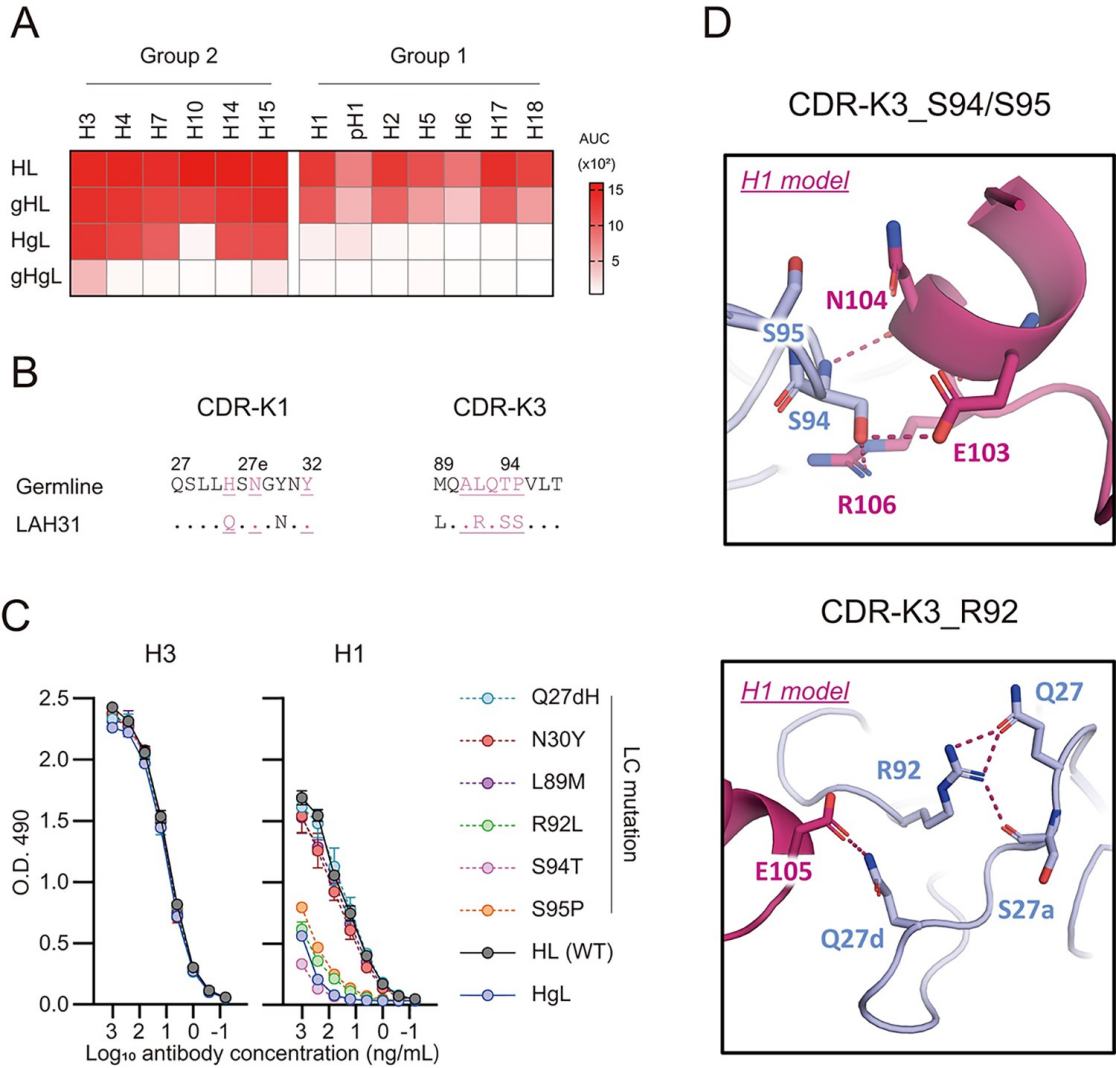
LAH31 acquires cross-group specificity primarily through light chain mutations. (A) Four types of LAH31 mAbs were created for bearing mutated heavy and light chains (HL), germline heavy and mutated light chains (gHL), mutated heavy and germline light chains (HgL), and germline heavy and light chains (gHgL). The binding of these LAH31 mAbs were assessed using ELISA with indicated HA subtypes. (B) Alignment of LAH31 CDR-K1 or CDR-K3 and its germline sequence. Contact residues are colored in pink and underlined. Dots indicate identical residues. CDR region is defined by IMGT, and residue positions are noted above the sequence according to Kabat numbering. (C) Contribution of individual light chain mutation was analyzed using ELISA against HAs of H3 X31 or H1 A/Narita/1/2009. Dilution curves of germline-reverted mAbs are indicated in dashed lines. The assay was conducted in duplicates and representative data from two independent experiments are represented. (D) Detailed recognition mode of the S94/S95 or R92 in CDR-K3 of LAH31 against H1 model epitope peptide. Peptide residues are indicated in H3 numbering.

IGHV1-69 encodes LAH31 heavy chain, which often acquires breadth by somatic hypermutations [35]. We addressed how the somatic hypermutations in heavy and light chains contribute to the cross-group breadth of LAH31. Heavy and light chains of LAH31 mAb were converted into germline forms, individually or simultaneously, and the binding of recombinant mAbs to the HA panel was evaluated using direct ELISA (Fig 5A). Germline conversion of LAH31 heavy chain retained the relatively normal breadth to group 1 HAs, whereas the germline conversion of light chain reduced the cross-group reactivity, highlighting a major contribution of mutated light chain on the cross-group breadth. By contrast, mutated heavy chain appeared to increase the reactivity to group 2 HAs, since pairing germline heavy and light chains reduced the reactivity to group 2 HA.

LAH31 light chain uses CDR-K1 and CDR-K3 for the epitope recognition (Fig 4A and S4 Table). CDR-K1 and CDR-K3 exhibit two and four replacement mutations, respectively (Fig 5B). Therefore, these six mutations were selected and converted into germline form individually in the recombinant LAH31 mAbs. Again, H3 reactivity was not affected by introducing six germline-converting mutations in light chain. In contrast, light chains with reverted mutation at 92, 94, and 95th position attenuated the H1-binding reactivity (Fig 5C), showing that these three residues are particularly important for acquiring cross-group breadth. Ser94 forms side-chain hydrogen bonds with Glu103 and Arg106 of peptide in the H1 modeling structure, which might contribute to this binding (Figs 4E and 5D). Thr94 in the germline causes steric hindrance and disrupting hydrogen bonds. Pro95 in the germline gene often assumes a cis peptide conformation, a typical signature of conventional canonical CDR-K3 structures [36]. Since LAH31 exhibited a mutation from Pro to Ser in this position, the conformational change of the main chain of CDR-K3 could have occurred, which compromised the hydrogen bond formations with the epitope.

Arg92 is not directly involved in antibody binding; however, it forms hydrogen bonds with amino acids in CDR-K1 (Fig 5D). This suggests that Arg92 might contribute to the structural stability of CDR-K1. Gln27d of the CDR-K1 forms hydrogen bonds with Glu105 in H1 subtype that is important for antibody-antigen interaction but is less determinant for the H3 subtype (Fig 4D and 4E). Therefore, these findings suggest that H1 subtype is strongly affected by the structural destabilization of CDR-K1 by the R92L mutation.

## Discussion

Large-scale conformational changes often occur in broadly neutralizing HA epitopes, such as CS [8, 37, 38], during the prefusion to postfusion conversion. Therefore, one rational approach for the universal vaccine design is to stabilize the antigen in the native prefusion state [9, 10]. However, several reports have highlighted the broadly protective properties of antibodies targeting occluded epitopes, that are inaccessible in a native HA conformation [11–14]. Therefore, there is a requirement for a better understanding of the antigenic nature of conserved epitopes concealed from antibodies in the native prefusion form, as well as of the mechanisms underlying breadth and protection achieved by the antibodies against such conserved epitopes. This could pave the way to a completely new, and more effective, vaccine design.

In this study, we conducted an in-depth analysis of the human LAH31 antibody, revealing unique characteristics of the LAH31 epitope and antibody. First, this epitope is only accessible after the postfusion conversion and exists in a unique KLH structure in the postfusion state. Second, this non-native epitope is exposed on infected cells in an accessible way to LAH31 antibody, which can mediate Fc-dependent clearance of the infected cells through ADCC, ADCP, or CDC [39]. Third, although LAH31 utilizes IGHV1-69 gene, characteristic of several broadly neutralizing antibody (bnAb) clones [20], it recognizes the epitope through the mutated light chain CDRs; a binding mode distinct from other IGHV1-69-type bnAbs [35].

While acquisition of cross-group breadth is exclusively dependent on the light chain mutations, the heavy chain gene usage, IGHV1-69, is also essential for molecular recognition of the LAH31. A characteristic feature of the IGHV1-69 gene is the germline-encoded hydrophobicity owing to hydrophobic residues at the tip of CDR-H2 (Phe or Leu at the 54th position), which contribute to direct contacts with hydrophobic conserved epitopes [20, 35]. The mutated LAH31 retains the hydrophobic interaction with Leu110 at the C-terminal region of the KLH epitope though replacement of the Leu54 with the hydrophobic Val. Additionally, germline encoded Arg50, adjacent to the CDR-H2 of LAH31, ensures a salt bridge with Glu103, a conserved residue of the KLH epitope across all HA subtypes (Fig 4C). Therefore, the initial recognition of LAH31 occurs in the C-terminus of the KLH region possibly in its germline encoded residues (Arg50 and hydrophobic Leu54) of heavy chain. LAH31 may also encompass conformational variability of the N-terminal region of the KLH epitope through hydrophilic interactions with the light chain CDRs (Figs 4A and S9), enabling cross-group breadth. This is in stark contrast to the CS-targeting IGHV1-69-type bnAbs, where most of the interactions with the hydrophobic stem groove are mediated by the heavy chain, with scarce contribution from the light chain [35]. Recently, head interface Abs and a novel class of bnAbs targeting the stem anchor epitope are shown to preferentially utilize specific light chain genes, highlighting a non-redundant role of the light chain for broad HA recognition [40–42]. Thus, the coordinated utility of both heavy and light chains, like LAH31, may potentiate the recognition of occluded epitopes which are hampered by multiple mechanisms.

An open and unanswered question pertains to how the non-native HA epitope is presented to B cells for clonal selection. Our previous study has shown that following pulmonary influenza virus infection, murine B cells targeting LAH epitopes are preferentially selected in lung germinal centers (GCs) [43]. Therefore, one may hypothesize that the postfusion-like HA antigens are presented locally by follicular dendritic cells (FDCs) within GCs. FDCs are known to recycle trapped antigens from the cell surface to endosomes [44]. These trapped HA antigens in FDCs may undergo endosomal acidification, leading to their conversion to postfusion-like structures. In line with this hypothesis of in vivo modification, the term “dark antigen” has been proposed as unknown, non-native antigens, resulting in the failure of immunogen recognition by approximately half of GC B cells [45]. Although the details of this process are still unknown, the identification of antibody clones that recognize epitopes unique to postfusion HAs, like the LAH31 non-native epitope, may help to dissect the antigenic structure involved in GC selection.

The structural basis how non-neutralizing LAH antibodies provide broad protection remains poorly understood. The activation of ADCC activity toward HA antigens on infected cells, yet not on HA-transfected cells, provided important insights on this subject. Since the elicitation of a broadly reactive B cell response, which is dominated by LAH repertoire, relies on viral replication [14, 46], HA expressed during the infection may be somewhat flexible to readily expose cryptic epitopes. One difference between infected and transfected cells, which may promote the conformational change of HA, is the cleavage of HA. Although uncleaved HA0 can modulate its structure, HA cleavage is necessary to complete the conversion to a postfusion state [47]. In addition, viral HA is metastable enough to mediate conformational changes even at neutral pH in unusual circumstances such as high temperature and urea treatment [48]. Thus, it could be possible that binding of LAH31 facilitates the postfusion conversion. However, given a strong ADCC activity against infected cells by FI6 (Fig 3E), prefusion HA and conformationally changed HA antigens appear to be concomitantly expressed. We speculate that infected cells present heterologous forms of HA antigens with distinct conformations, allowing antibody recognition of CS epitopes on prefusion HA, or LAH epitope on non-native HA.

Besides influenza virus antigen, class 1 viral envelope proteins (HIV-1 Env, RSV fusion, SARS-CoV-2 spike, etc.) undergo drastic structural alternations from prefusion to postfusion state [49–51]. This may pose as an increased opportunity of antibody recognition of hidden yet vulnerable epitopes. Antibodies induced by postfusion antigens have been considered less potent or even harmful for humans, according to the clinical study of RSV vaccine [52, 53]. Previous studies using pig or ferret models immunized with mismatched H1N2 vaccine observed the correlation between vaccine-associated enhanced respiratory disease and the levels of fusion-promoting antibodies against HA2 region close to fusion peptide [54, 55]. However, LAH epitopes are different from this HA2 epitopes for fusion-promoting antibodies and the antibodies induced by postfusion HA antigens did not enhance respiratory disease at least in mouse model [14]. Further understanding the mechanistic insights of the antigenic conversion and antibody recognition may pave the way for the next generation of universal vaccine design, to elicit immune responses against well-conserved hidden epitopes.

## Materials and methods

### Ethics statement

All animal experiments were approved by the Animal Ethics Committee of the National Institute of Infectious Diseases, Japan (approved numbers: 121116, 122088, and 122123) and performed in accordance with the guidelines of the Institutional Animal Care and Use Committee.

### Mice and viruses

C57BL/6 mice were purchased from Japan SLC. All mice were maintained under specific pathogen free condition and female were used at ages of 7–12 weeks.

The X31 (H3N2) viruses were propagated on embryonated chicken eggs and purified using a 10–50% sucrose gradient as previously described [56]. The A/Guangdong-Maonan/SWL1536/2019 (H1N1) virus was kindly provided by Dr. Hideki Asanuma (National Institute of Infectious Diseases, Japan).

### Synthetic LAH peptides and recombinant proteins

The LAH peptides used for ELISA were synthesized commercially (GL Biochem or Genscript). The full-length peptide of LAH (amino acids: 76–130) in A/Hong Kong/1/68 (H3N2) HA2 was designed with acetylation of the N-terminus and the addition of a Flag tag (DYKDDDDK) and a cysteine at the C-terminus, as previously described [57]. The 30- or 15-residue overlapping LAH peptides were designed with amidation to the C-terminus and biotinylation to the N-terminus. The 30-residue peptides were further added a 2× GGGS tag to the N-terminus. For crystallography, the 15-residue LAH peptide (VALENQHTIDLTDSE) without modification was commercially synthesized (Eurofins Genomics).

The human codon-optimized nucleotide sequences, encoding for the HA ectodomain, were commercially synthesized (Eurofins Genomics) and subcloned into the mammalian expression vector pCMV with a 6× His tag and the Avitag at the C-terminus. To express the trimeric full-length HA, the foldon trimerization domain of T4 fibritin, with or without a thrombin cleavage site and the Strep tag II, was further fused upstream of a 6× His tag. To express postfusion HA2 antigen, a part of the sequence of EHA2 [28], which is designed with extracting fusion peptide and substitution of Cys137 to Ser in X31 HA2 (amino acids: 24–185), was subcloned into pCMV with the foldon, 6× His tag and Avitag at the C-terminus.

All HA and HA2 constructs were transfected into Expi293F cells (Thermo Fisher Scientific) as previously described [58, 59]. Cell supernatants were collected 5~7 days after transfection. The proteins were purified with TALON Metal Affinity Resin (Takara) or HisTrap excel column (Cytiva), and then subjected to further analysis after dialysis against PBS.

### Amplification of V(D)J rearrangements

The V(D)J sequence of LAH31 was amplified from single-cell cultured, memory B cells obtained previously [14]. V(D)J usage, germline sequence, and number of somatic hypermutations were identified by IgBlast IMGT as previously described [60]. Sequences data for LAH31 have been deposited in GenBank (accession numbers: OQ442300, OQ442301).

### Generation of monoclonal antibodies

V_H_/V_L_ genes of somatically mutated, germline-reverted LAH31, or published mAbs, were subcloned into human IgG1 and human Igκ expression vectors. For in vivo experiments and in vitro functional assays, we generated musinized antibodies through the replacement of the variable region genes into mouse IgG2c wild-type, KA-, LALA-, or LALA-PG mutant and mouse Igκ expression vectors. To generate LAH31 Fab fragments, V_H_ gene of LAH31 was introduced into a human Fab expression vector (a gift of Dr. Aaron G Schmidt, Harvard Medical School). All antibody constructs were transfected into Expi293F cells (Thermo Fisher Scientific). Cell supernatants were collected 4–5 days later. The IgG and Fab antibodies were purified using protein G column (Cytiva) and TALON Metal Affinity Resin (Takara), respectively, and then subjected to further analysis after dialysis against PBS.

### ELISA

ELISA plates (96-well) were coated with antigens at a concentration of 5 μg/ml. For the analysis of H1–H18 subtypes of trimeric HAs, 384-well ELISA plates were used. For the coating with the 15-residue overlapping LAH peptides, prefusion HA or postfusion HA2, the plates were pre-coated with recombinant streptavidin (Wako) at 10, 0.5 and 1 μg/ml, respectively. For the coating with the commercial influenza split HA vaccine of 2022–2023 season (Daiichi Sankyo Co., Ltd.), the plates were pre-coated with S5V2-29 [12] at 6 μg/ml. After blocking with PBS containing 1% bovine serum albumin, serially-diluted mAbs were applied to the plates, and then incubated with goat anti-human IgG-HRP, goat anti-mouse IgG-HRP, or goat anti-mouse IgG2c-HRP (Southern Biotech). For the plates pre-coated with streptavidin or S5V2-29, an additional coating with the antigens were conducted at 5 μg/ml after blocking, and the protocol was followed as described above. HRP-activity was visualized with an OPD substrate (Sigma-Aldrich), and OD490 was measured using an iMark Microplate Reader (Bio-Rad).

For the analysis of polyreactivity, ELISA plates were coated with either of 10 μg/ml dsDNA of calf thymus (Worthington Biochemical Corporation), 5 μg/ml insulin from bovine pancreas, or 10 μg/ml LPS of E.coli O111:B4 (Sigma-Aldrich). For dsDNA, poly-L-lysine (Wako) was pre-coated at 10 μg/ml. After blocking, the protocol was followed as described above. 72H11 [24] (a gift of Dr. Tomohiro Kurosaki, Osaka University, Japan) was applied as a positive control.

### Crystallization and data collection

The LAH31 Fab was mixed with the LAH peptide in a 1:5 molar ratio at a protein concentration of 200 μM. The mixture was used for crystallization through the sitting-drop vapor diffusion method at 293 K. Crystals of the LAH31-LAH peptide complex were obtained in the reservoir solution containing 0.1 M HEPES–NaOH (pH 7.5), 0.2 M ammonium formate, and 16% PEG 3350. The crystals were then cryo-protected with the reservoir solution plus 25% glycerol. Diffraction data sets were collected using a beamline BL17A at Photon Factory (Ibaraki, Japan). Collected data were indexed, integrated, scaled, and merged using XDS [61].

### Structure determination and refinement

The structure of the LAH31-LAH peptide complex was determined by the molecular replacement method with Phaser [62], using the previously reported light chain of PI3-e12 Fab (PDB: 6wrp) and heavy chain of IGHV1-69 germline antibody with CDR H3 sequence of CR9114 (PDB: 5wl2) as search models. The initial protein models were rebuilt using Phenix AutoBuild [63] and fitted manually using Coot [64]. The structure was then refined using phenix.refine [65]. The data collection and refinement statistics are summarized in S2 Table. Coordinates and structure factors have been deposited in the Protein Data Bank (PDB: 8ib1). Figures were prepared using PyMOL (Schrödinger) or UCSF Chimera [66].

### Biolayer interferometry

The kinetics of LAH31 Fab binding to prefusion HA or postfusion HA2 was determined using an Octet R8 system (Sartorius) at 30°C with shaking at 1,000 rpm. The biotinylated prefusion HA or postfusion HA2 were loaded at 0.664 μg/mL and 0.4 μg/mL in 1× kinetics buffer (Sartorius) for 300 s onto streptavidin biosensors (Sartorius), respectively, and incubated with serially diluted LAH31 Fab (2-fold derail dilution start from 8,000 nM and 80 nM for prefusion HA and postfusion HA2, respectively) for 180 s, followed by immersion in 1× kinetics buffer for 300 s of dissociation time.

### Computational modeling of antibody-peptide interactions

Energetics and hydrogen bonding capability in the interaction between LAH31 Fab and LAH peptides were assessed through the FlexPepDock refinement protocol [67] in Rosetta release 3.13 [68]. The starting structures of the docking simulations were prepared either from our crystal structure of the LAH31-LAH peptide complex (H3 strain) or from the complex with the derived peptides, where the original peptide (H3 strain) in the crystal structure was computationally mutated to the one in each strain (H1/H17/H18 or H8/H9/H12 strain). In silico alanine scanning was performed using the Flex-ddG protocol [69] based on the crystal structures (H3 strain) or the docked complexes (H1/H17/H18 or H8/H9/H12 strain). Throughout the calculations, we employed the REF2015 scoring function [70].

### In vivo protection experiments

C57BL/6 mice were intraperitoneally (i.p.) treated with 5 mg/kg body weight of murine IgG2c wild-type or Fc-mutant antibodies. To evaluate dose dependency, some mice were i.p. treated with either 2.5 or 1.25 mg/kg of LAH31 IgG2c. Three hours later, mice were anesthetized by i.p. injection with a mixture of 3% medetomidine hydrochloride (Zenoaq), 10% butorphanol tartrate (Meiji Seika Pharma Co.), and 8% midazolam (Astellas Pharma Inc.) in a final volume of 200 μl. The mice were then intranasally (i.n.) infected with X31 (H3N2) or A/Guangdong-Maonan/SWL1536/2019 (H1N1) virus at a dose of 5 mLD_50_ in a final volume of 50 μl, followed by i.p. injection of 100 μl antagonist containing 2.4% atipamezole hydrochloride (Zenoaq). All mice were monitored daily for survival and body weight during 2 weeks after infection. The humane endpoint was set at 25% body weight loss relative to the initial body weight at the time of infection.

### In vitro neutralization assay

Virus-neutralization antibody titers were determined using microneutralization assays with the MDCK cell line (Influenza Virus Research Center, National Institute of Infectious Diseases, Japan). Serially diluted mAbs were pre-incubated with X31 (100 TCID_50_), and then added to MDCK cells in the presence of 5 μg/ml acetyltrypsin (Sigma). After 3 days of incubation, the virus-neutralization antibody titer was determined based on the CPE and expressed as the minimal antibody concentration that inhibits viral replication.

### Mouse ADCC reporter assay using virus-infected or HA-expressing cells

To obtain virus-infected cell line, MDCK cells were plated at a density of 2.5 × 10^4^ cells/well in a white flat-bottom 96-well assay plates (Corning) and incubated overnight in a 37°C 5% CO2 incubator. The following day, cells were washed once with PBS and once with OptiMEM (Gibco), and then incubated with X31 (MOI 3) in the presence of 5 μg/ml acetyltrypsin (Sigma) overnight at 34°C.

The X31 HA-expressing EL4 cells were kindly provided by Dr. Garnett Kelsoe (Duke University School of Medicine), and were maintained as previously described [12].

ADCC activity was determined using a mouse ADCC reporter bioassay kit (Promega) according to manufacturer’s instructions with minor modifications. Briefly, serially diluted mAbs were pre-incubated with the target cell lines for 15 minutes, followed by the addition of effector cells at an effector/target ratio of 3:1. After 6 hours of incubation at 37°C, Bio-Glo Reagent was added for a 30 minutes incubation in the infected condition and for a 15 minutes incubation in HA-expressing condition at room temperature and luminescence was measured by GloMax Navigator Microplate Luminometer (Promega). Irrelevant SARS-CoV-2 specific antibody B38 [71] was used as a negative control.

### Statistical analyses

Statistical analyses were conducted using a two-tailed Mann–Whitney U test. Statistical significance between the body weight loss was determined using a two-way ANOVA test. Statistical significance between the survival rates was determined by Kaplan–Meier survival curves and log-rank test. All statistical analyses for in vivo studies were performed using Prism (GraphPad). *P* values < 0.05 were considered statistically significant and indicated by asterisks: **p < 0.01, ***p < 0.001, ****p < 0.0001, †*p* < 0.05, ††††*p* < 0.0001. For multi-group comparisons of survival curves, statistical significance was adjusted by Bonferroni correction. Corrected alpha values and corresponding asterisks are denoted in figure legends.

## Supporting information

S1 Fig Binding ability of LAH31 against common self-antigens. The binding ability of LAH31 against dsDNA, insulin or LPS was assessed using ELISA. FI6 and 72H11 were used for comparison and as a positive control, respectively. The Assay was conducted in duplicates and representative data from two independent experiments are represented as mean ± SD.
(TIF)

S2 Fig Neutralizing activity of LAH31. Serially diluted mAbs were applied to the virus-neutralization assay using X31 virus. FI6 was used as a positive control. Minimal neutralizing titer is shown (100 μg/ml as maximum). Bars represent the mean.
(TIF)

S3 Fig Validation of LAH31 Fc mutants in binding to HA. The binding ability of WT and Fc mutated (KA, LALA, LALA-PG) LAH31 against X31 HA was assessed using ELISA. The Assay was conducted in duplicates and representative data from two independent experiments are represented as mean ± SD.
(TIF)

S4 Fig Binding ability of LAH31 against commercial influenza vaccine. The binding ability of LAH31 against commercial influenza split vaccine (2022–2023 season) was assessed using ELISA. The vaccine antigens were captured by pre-coated S5V2-29. FI6 was used as a positive control. The Assay was conducted in triplicates and representative data from two independent experiments are represented as mean ± SD.
(TIF)

S5 Fig Binding ability of LAH31 against prefusion HA and postfusion HA2 in ELISA. LAH31 and published mAbs with different epitope specificity (FI6, CS; S5V2-29, HI; F045-092, RBS) were tested for binding to X31 (H3N2) prefusion HA (top) or postfusion HA2 (bottom). For each antigen, ELISA was performed by coating directly (left) or via-streptavidin (right). The Assay was conducted in duplicates and representative data from two independent experiments are represented as mean ± SD.
(TIF)

S6 Fig Phylogenetic tree of IAV HAs. Each node is colored based on whether LAH31 binds to (pink, group 1; grey, group 2) or not (blue). Subtypes reported to be infectious to human are underlined.
(TIF)

S7 Fig Interface energy between LAH31 and epitope LAH peptide calculated by in silico docking simulation. 200 model structures of complex in each subtype were produced from experimental crystal structure and the calculated energy of 10 models with highest scoring are plotted. The *P* values were determined with two-tailed Mann–Whitney U test. Not significant (ns), **p < 0.01.
(TIF)

S8 Fig Alanine scanning of epitope peptide region in H3 simulated from model structure. The structure prepared in S7 Fig with the highest scoring was chosen. Changes of free energy in LAH31 binding were calculated by virtually replacing corresponding residue to Ala and are indicated as a bar graph. The individual amino acid sequences are noted below each bar.
(TIF)

S9 Fig Comparison of LAH31 epitope region between H1 and H3 postfusion HA2. Structure of postfusion HA2 in H3 X31 (grey: 1qu1) and H1 A/Luxembourg/43/2009 (blue: 6gol) were overlayed (left). Kinked loop-helix region is further zoomed in (right).
(TIF)

S1 Table Viral strains of HA used in this study. (DOC)

S2 Table Data collection and refinement statistics. (DOC)

S3 Table Sequence of HA2 region used for postfusion HA2 antigen in this study. The residue number and amino acid sequence of HA2 region of H3 X31 HA used in this study is shown. Residues are numbered in H3 numbering. LAH region is highlighted by bold capital and underline in the sequence.
(DOC)

S4 Table Contact residues located within 4Å distance between LAH31 and epitope LAH peptide. (DOC)

## References

[1] Wu NC, Wilson IA. Structural Biology of Influenza Hemagglutinin: An Amaranthine Adventure. Viruses. 2020;12(9). Epub 20200922. doi: 10.3390/v12091053 ; PubMed Central PMCID: PMC7551194. 32971825 PMC7551194

[2] Tong S, Zhu X, Li Y, Shi M, Zhang J, Bourgeois M, et al. New world bats harbor diverse influenza A viruses. PLoS Pathog. 2013;9(10):e1003657. Epub 20131010. doi: 10.1371/journal.ppat.1003657 ; PubMed Central PMCID: PMC3794996. 24130481 PMC3794996

[3] Tong S, Li Y, Rivailler P, Conrardy C, Castillo DA, Chen LM, et al. A distinct lineage of influenza A virus from bats. Proc Natl Acad Sci U S A. 2012;109(11):4269–74. Epub 20120227. doi: 10.1073/pnas.1116200109 ; PubMed Central PMCID: PMC3306675. 22371588 PMC3306675

[4] Nobusawa E, Aoyama T, Kato H, Suzuki Y, Tateno Y, Nakajima K. Comparison of complete amino acid sequences and receptor-binding properties among 13 serotypes of hemagglutinins of influenza A viruses. Virology. 1991;182(2):475–85. doi: 10.1016/0042-6822(91)90588-3 . 2024485

[5] Kanekiyo M, Graham BS. Next-Generation Influenza Vaccines. Cold Spring Harb Perspect Med. 2021;11(8). Epub 20210802. doi: 10.1101/cshperspect.a038448 ; PubMed Central PMCID: PMC8327825. 32229612 PMC8327825

[6] Sun X, Ling Z, Yang Z, Sun B. Broad neutralizing antibody-based strategies to tackle influenza. Curr Opin Virol. 2022;53:101207. Epub 20220205. doi: 10.1016/j.coviro.2022.101207 . 35131735

[7] Ohshima N, Iba Y, Kubota-Koketsu R, Asano Y, Okuno Y, Kurosawa Y. Naturally occurring antibodies in humans can neutralize a variety of influenza virus strains, including H3, H1, H2, and H5. J Virol. 2011;85(21):11048–57. Epub 20110824. doi: 10.1128/JVI.05397-11 ; PubMed Central PMCID: PMC3194982. 21865387 PMC3194982

[8] Corti D, Voss J, Gamblin SJ, Codoni G, Macagno A, Jarrossay D, et al. A neutralizing antibody selected from plasma cells that binds to group 1 and group 2 influenza A hemagglutinins. Science. 2011;333(6044):850–6. Epub 20110728. doi: 10.1126/science.1205669 . 21798894

[9] Yassine HM, Boyington JC, McTamney PM, Wei CJ, Kanekiyo M, Kong WP, et al. Hemagglutinin-stem nanoparticles generate heterosubtypic influenza protection. Nat Med. 2015;21(9):1065–70. Epub 20150824. doi: 10.1038/nm.3927 . 26301691

[10] Impagliazzo A, Milder F, Kuipers H, Wagner MV, Zhu X, Hoffman RM, et al. A stable trimeric influenza hemagglutinin stem as a broadly protective immunogen. Science. 2015;349(6254):1301–6. Epub 20150824. doi: 10.1126/science.aac7263 . 26303961

[11] Bangaru S, Lang S, Schotsaert M, Vanderven HA, Zhu X, Kose N, et al. A Site of Vulnerability on the Influenza Virus Hemagglutinin Head Domain Trimer Interface. Cell. 2019;177(5):1136–52 e18. doi: 10.1016/j.cell.2019.04.011 ; PubMed Central PMCID: PMC6629437. 31100268 PMC6629437

[12] Watanabe A, McCarthy KR, Kuraoka M, Schmidt AG, Adachi Y, Onodera T, et al. Antibodies to a Conserved Influenza Head Interface Epitope Protect by an IgG Subtype-Dependent Mechanism. Cell. 2019;177(5):1124–35 e16. doi: 10.1016/j.cell.2019.03.048 ; PubMed Central PMCID: PMC6825805. 31100267 PMC6825805

[13] Bajic G, Maron MJ, Adachi Y, Onodera T, McCarthy KR, McGee CE, et al. Influenza Antigen Engineering Focuses Immune Responses to a Subdominant but Broadly Protective Viral Epitope. Cell Host Microbe. 2019;25(6):827–35 e6. Epub 20190516. doi: 10.1016/j.chom.2019.04.003 ; PubMed Central PMCID: PMC6748655. 31104946 PMC6748655

[14] Adachi Y, Tonouchi K, Nithichanon A, Kuraoka M, Watanabe A, Shinnakasu R, et al. Exposure of an occluded hemagglutinin epitope drives selection of a class of cross-protective influenza antibodies. Nat Commun. 2019;10(1):3883. Epub 20190828. doi: 10.1038/s41467-019-11821-6 ; PubMed Central PMCID: PMC6713747. 31462639 PMC6713747

[15] Das DK, Govindan R, Nikic-Spiegel I, Krammer F, Lemke EA, Munro JB. Direct Visualization of the Conformational Dynamics of Single Influenza Hemagglutinin Trimers. Cell. 2018;174(4):926–37 e12. Epub 20180628. doi: 10.1016/j.cell.2018.05.050 ; PubMed Central PMCID: PMC6086748. 29961575 PMC6086748

[16] Benton DJ, Gamblin SJ, Rosenthal PB, Skehel JJ. Structural transitions in influenza haemagglutinin at membrane fusion pH. Nature. 2020;583(7814):150–3. Epub 20200527. doi: 10.1038/s41586-020-2333-6 ; PubMed Central PMCID: PMC7116728. 32461688 PMC7116728

[17] Bullough PA, Hughson FM, Skehel JJ, Wiley DC. Structure of influenza haemagglutinin at the pH of membrane fusion. Nature. 1994;371(6492):37–43. doi: 10.1038/371037a0 . 8072525

[18] Henry Dunand CJ, Leon PE, Huang M, Choi A, Chromikova V, Ho IY, et al. Both Neutralizing and Non-Neutralizing Human H7N9 Influenza Vaccine-Induced Monoclonal Antibodies Confer Protection. Cell Host Microbe. 2016;19(6):800–13. doi: 10.1016/j.chom.2016.05.014 ; PubMed Central PMCID: PMC4901526. 27281570 PMC4901526

[19] Wada Y, Nithichanon A, Nobusawa E, Moise L, Martin WD, Yamamoto N, et al. A humanized mouse model identifies key amino acids for low immunogenicity of H7N9 vaccines. Sci Rep. 2017;7(1):1283. Epub 20170428. doi: 10.1038/s41598-017-01372-5 ; PubMed Central PMCID: PMC5430863. 28455520 PMC5430863

[20] Chen F, Tzarum N, Wilson IA, Law M. V(H)1-69 antiviral broadly neutralizing antibodies: genetics, structures, and relevance to rational vaccine design. Curr Opin Virol. 2019;34:149–59. Epub 20190316. doi: 10.1016/j.coviro.2019.02.004 ; PubMed Central PMCID: PMC7266006. 30884330 PMC7266006

[21] Brouwer PJM, Caniels TG, van der Straten K, Snitselaar JL, Aldon Y, Bangaru S, et al. Potent neutralizing antibodies from COVID-19 patients define multiple targets of vulnerability. Science. 2020;369(6504):643–50. Epub 20200615. doi: 10.1126/science.abc5902 ; PubMed Central PMCID: PMC7299281. 32540902 PMC7299281

[22] Sangesland M, Torrents de la Pena A, Boyoglu-Barnum S, Ronsard L, Mohamed FAN, Moreno TB, et al. Allelic polymorphism controls autoreactivity and vaccine elicitation of human broadly neutralizing antibodies against influenza virus. Immunity. 2022;55(9):1693–709 e8. Epub 20220810. doi: 10.1016/j.immuni.2022.07.006 ; PubMed Central PMCID: PMC9474600. 35952670 PMC9474600

[23] Bajic G, van der Poel CE, Kuraoka M, Schmidt AG, Carroll MC, Kelsoe G, et al. Autoreactivity profiles of influenza hemagglutinin broadly neutralizing antibodies. Sci Rep. 2019;9(1):3492. Epub 20190305. doi: 10.1038/s41598-019-40175-8 ; PubMed Central PMCID: PMC6401307. 30837606 PMC6401307

[24] Sakakibara S, Arimori T, Yamashita K, Jinzai H, Motooka D, Nakamura S, et al. Clonal evolution and antigen recognition of anti-nuclear antibodies in acute systemic lupus erythematosus. Sci Rep. 2017;7(1):16428. Epub 20171127. doi: 10.1038/s41598-017-16681-y ; PubMed Central PMCID: PMC5703881. 29180749 PMC5703881

[25] Idusogie EE, Presta LG, Gazzano-Santoro H, Totpal K, Wong PY, Ultsch M, et al. Mapping of the C1q binding site on rituxan, a chimeric antibody with a human IgG1 Fc. J Immunol. 2000;164(8):4178–84. doi: 10.4049/jimmunol.164.8.4178 . 10754313

[26] Arduin E, Arora S, Bamert PR, Kuiper T, Popp S, Geisse S, et al. Highly reduced binding to high and low affinity mouse Fc gamma receptors by L234A/L235A and N297A Fc mutations engineered into mouse IgG2a. Mol Immunol. 2015;63(2):456–63. Epub 20141018. doi: 10.1016/j.molimm.2014.09.017 . 25451975

[27] Lo M, Kim HS, Tong RK, Bainbridge TW, Vernes JM, Zhang Y, et al. Effector-attenuating Substitutions That Maintain Antibody Stability and Reduce Toxicity in Mice. J Biol Chem. 2017;292(9):3900–8. Epub 20170111. doi: 10.1074/jbc.M116.767749 ; PubMed Central PMCID: PMC5339770. 28077575 PMC5339770

[28] Chen J, Skehel JJ, Wiley DC. N- and C-terminal residues combine in the fusion-pH influenza hemagglutinin HA(2) subunit to form an N cap that terminates the triple-stranded coiled coil. Proc Natl Acad Sci U S A. 1999;96(16):8967–72. doi: 10.1073/pnas.96.16.8967 ; PubMed Central PMCID: PMC17716. 10430879 PMC17716

[29] Mavor D, Barlow KA, Asarnow D, Birman Y, Britain D, Chen W, et al. Extending chemical perturbations of the ubiquitin fitness landscape in a classroom setting reveals new constraints on sequence tolerance. Biol Open. 2018;7(7). Epub 20180723. doi: 10.1242/bio.036103 ; PubMed Central PMCID: PMC6078352. 30037883 PMC6078352

[30] Tam C, Kukimoto-Niino M, Miyata-Yabuki Y, Tsuda K, Mishima-Tsumagari C, Ihara K, et al. Targeting Ras-binding domain of ELMO1 by computational nanobody design. Commun Biol. 2023;6(1):284. Epub 20230317. doi: 10.1038/s42003-023-04657-w ; PubMed Central PMCID: PMC10023680. 36932164 PMC10023680

[31] Chen D, Bali S, Singh R, Wosztyl A, Mullapudi V, Vaquer-Alicea J, et al. FTD-tau S320F mutation stabilizes local structure and allosterically promotes amyloid motif-dependent aggregation. Nat Commun. 2023;14(1):1625. Epub 20230323. doi: 10.1038/s41467-023-37274-6 ; PubMed Central PMCID: PMC10036635. 36959205 PMC10036635

[32] Baker AT, Boyd RJ, Sarkar D, Teijeira-Crespo A, Chan CK, Bates E, et al. ChAdOx1 interacts with CAR and PF4 with implications for thrombosis with thrombocytopenia syndrome. Sci Adv. 2021;7(49):eabl8213. Epub 20211201. doi: 10.1126/sciadv.abl8213 ; PubMed Central PMCID: PMC8635433. 34851659 PMC8635433

[33] Barnes JE, Lund-Andersen PK, Patel JS, Ytreberg FM. The effect of mutations on binding interactions between the SARS-CoV-2 receptor binding domain and neutralizing antibodies B38 and CB6. Sci Rep. 2022;12(1):18819. Epub 20221105. doi: 10.1038/s41598-022-23482-5 ; PubMed Central PMCID: PMC9637166. 36335244 PMC9637166

[34] Lu IN, Kirsteina A, Farinelle S, Willieme S, Tars K, Muller CP, et al. Structure and applications of novel influenza HA tri-stalk protein for evaluation of HA stem-specific immunity. PLoS One. 2018;13(9):e0204776. Epub 20180927. doi: 10.1371/journal.pone.0204776 ; PubMed Central PMCID: PMC6160157. 30261065 PMC6160157

[35] Pappas L, Foglierini M, Piccoli L, Kallewaard NL, Turrini F, Silacci C, et al. Rapid development of broadly influenza neutralizing antibodies through redundant mutations. Nature. 2014;516(7531):418–22. Epub 20141005. doi: 10.1038/nature13764 . 25296253

[36] Kuroda D, Shirai H, Kobori M, Nakamura H. Systematic classification of CDR-L3 in antibodies: implications of the light chain subtypes and the VL-VH interface. Proteins. 2009;75(1):139–46. doi: 10.1002/prot.22230 . 18798566

[37] Kallewaard NL, Corti D, Collins PJ, Neu U, McAuliffe JM, Benjamin E, et al. Structure and Function Analysis of an Antibody Recognizing All Influenza A Subtypes. Cell. 2016;166(3):596–608. Epub 20160721. doi: 10.1016/j.cell.2016.05.073 ; PubMed Central PMCID: PMC4967455. 27453466 PMC4967455

[38] Dreyfus C, Laursen NS, Kwaks T, Zuijdgeest D, Khayat R, Ekiert DC, et al. Highly conserved protective epitopes on influenza B viruses. Science. 2012;337(6100):1343–8. Epub 20120809. doi: 10.1126/science.1222908 ; PubMed Central PMCID: PMC3538841. 22878502 PMC3538841

[39] Boudreau CM, Alter G. Extra-Neutralizing FcR-Mediated Antibody Functions for a Universal Influenza Vaccine. Front Immunol. 2019;10:440. Epub 20190318. doi: 10.3389/fimmu.2019.00440 ; PubMed Central PMCID: PMC6436086. 30949165 PMC6436086

[40] Guthmiller JJ, Han J, Utset HA, Li L, Lan LY, Henry C, et al. Broadly neutralizing antibodies target a haemagglutinin anchor epitope. Nature. 2022;602(7896):314–20. Epub 20211223. doi: 10.1038/s41586-021-04356-8 ; PubMed Central PMCID: PMC8828479. 34942633 PMC8828479

[41] Zost SJ, Dong J, Gilchuk IM, Gilchuk P, Thornburg NJ, Bangaru S, et al. Canonical features of human antibodies recognizing the influenza hemagglutinin trimer interface. J Clin Invest. 2021;131(15). doi: 10.1172/JCI146791 ; PubMed Central PMCID: PMC8321569. 34156974 PMC8321569

[42] McCarthy KR, Lee J, Watanabe A, Kuraoka M, Robinson-McCarthy LR, Georgiou G, et al. A Prevalent Focused Human Antibody Response to the Influenza Virus Hemagglutinin Head Interface. mBio. 2021;12(3):e0114421. Epub 20210601. doi: 10.1128/mBio.01144-21 ; PubMed Central PMCID: PMC8262862. 34060327 PMC8262862

[43] Adachi Y, Onodera T, Yamada Y, Daio R, Tsuiji M, Inoue T, et al. Distinct germinal center selection at local sites shapes memory B cell response to viral escape. J Exp Med. 2015;212(10):1709–23. Epub 20150831. doi: 10.1084/jem.20142284 ; PubMed Central PMCID: PMC4577849. 26324444 PMC4577849

[44] Heesters BA, Chatterjee P, Kim YA, Gonzalez SF, Kuligowski MP, Kirchhausen T, et al. Endocytosis and recycling of immune complexes by follicular dendritic cells enhances B cell antigen binding and activation. Immunity. 2013;38(6):1164–75. Epub 20130613. doi: 10.1016/j.immuni.2013.02.023 ; PubMed Central PMCID: PMC3773956. 23770227 PMC3773956

[45] Kuraoka M, Schmidt AG, Nojima T, Feng F, Watanabe A, Kitamura D, et al. Complex Antigens Drive Permissive Clonal Selection in Germinal Centers. Immunity. 2016;44(3):542–52. Epub 20160303. doi: 10.1016/j.immuni.2016.02.010 ; PubMed Central PMCID: PMC4794380. 26948373 PMC4794380

[46] Miyauchi K, Adachi Y, Tonouchi K, Yajima T, Harada Y, Fukuyama H, et al. Influenza virus infection expands the breadth of antibody responses through IL-4 signalling in B cells. Nat Commun. 2021;12(1):3789. Epub 20210618. doi: 10.1038/s41467-021-24090-z ; PubMed Central PMCID: PMC8213721. 34145279 PMC8213721

[47] Garcia-Moro E, Zhang J, Calder LJ, Brown NR, Gamblin SJ, Skehel JJ, et al. Reversible structural changes in the influenza hemagglutinin precursor at membrane fusion pH. Proc Natl Acad Sci U S A. 2022;119(33):e2208011119. Epub 20220808. doi: 10.1073/pnas.2208011119 ; PubMed Central PMCID: PMC9388137. 35939703 PMC9388137

[48] Carr CM, Chaudhry C, Kim PS. Influenza hemagglutinin is spring-loaded by a metastable native conformation. Proc Natl Acad Sci U S A. 1997;94(26):14306–13. doi: 10.1073/pnas.94.26.14306 ; PubMed Central PMCID: PMC24954. 9405608 PMC24954

[49] Battles MB, McLellan JS. Respiratory syncytial virus entry and how to block it. Nat Rev Microbiol. 2019;17(4):233–45. doi: 10.1038/s41579-019-0149-x ; PubMed Central PMCID: PMC7096974. 30723301 PMC7096974

[50] Jackson CB, Farzan M, Chen B, Choe H. Mechanisms of SARS-CoV-2 entry into cells. Nat Rev Mol Cell Biol. 2022;23(1):3–20. Epub 20211005. doi: 10.1038/s41580-021-00418-x ; PubMed Central PMCID: PMC8491763. 34611326 PMC8491763

[51] Xiao T, Cai Y, Chen B. HIV-1 Entry and Membrane Fusion Inhibitors. Viruses. 2021;13(5). Epub 20210423. doi: 10.3390/v13050735 ; PubMed Central PMCID: PMC8146413. 33922579 PMC8146413

[52] Chang LA, Phung E, Crank MC, Morabito KM, Villafana T, Dubovsky F, et al. A prefusion-stabilized RSV F subunit vaccine elicits B cell responses with greater breadth and potency than a postfusion F vaccine. Sci Transl Med. 2022;14(676):eade0424. Epub 20221221. doi: 10.1126/scitranslmed.ade0424 . 36542692 PMC11345946

[53] Kim HW, Canchola JG, Brandt CD, Pyles G, Chanock RM, Jensen K, et al. Respiratory syncytial virus disease in infants despite prior administration of antigenic inactivated vaccine. Am J Epidemiol. 1969;89(4):422–34. doi: 10.1093/oxfordjournals.aje.a120955 . 4305198

[54] Khurana S, Loving CL, Manischewitz J, King LR, Gauger PC, Henningson J, et al. Vaccine-induced anti-HA2 antibodies promote virus fusion and enhance influenza virus respiratory disease. Sci Transl Med. 2013;5(200):200ra114. doi: 10.1126/scitranslmed.3006366 . 23986398

[55] Kimble JB, Wymore Brand M, Kaplan BS, Gauger P, Coyle EM, Chilcote K, et al. Vaccine-Associated Enhanced Respiratory Disease following Influenza Virus Infection in Ferrets Recapitulates the Model in Pigs. J Virol. 2022;96(5):e0172521. Epub 20220105. doi: 10.1128/JVI.01725-21 ; PubMed Central PMCID: PMC8906406. 34985999 PMC8906406

[56] Takahashi Y, Hasegawa H, Hara Y, Ato M, Ninomiya A, Takagi H, et al. Protective immunity afforded by inactivated H5N1 (NIBRG-14) vaccine requires antibodies against both hemagglutinin and neuraminidase in mice. J Infect Dis. 2009;199(11):1629–37. doi: 10.1086/598954 . 19385735

[57] Wang TT, Tan GS, Hai R, Pica N, Ngai L, Ekiert DC, et al. Vaccination with a synthetic peptide from the influenza virus hemagglutinin provides protection against distinct viral subtypes. Proc Natl Acad Sci U S A. 2010;107(44):18979–84. Epub 20101018. doi: 10.1073/pnas.1013387107 ; PubMed Central PMCID: PMC2973924. 20956293 PMC2973924

[58] Onodera T, Kita S, Adachi Y, Moriyama S, Sato A, Nomura T, et al. A SARS-CoV-2 antibody broadly neutralizes SARS-related coronaviruses and variants by coordinated recognition of a virus-vulnerable site. Immunity. 2021;54(10):2385–98 e10. Epub 20210824. doi: 10.1016/j.immuni.2021.08.025 ; PubMed Central PMCID: PMC8382582. 34508662 PMC8382582

[59] Saito S, Sano K, Suzuki T, Ainai A, Taga Y, Ueno T, et al. IgA tetramerization improves target breadth but not peak potency of functionality of anti-influenza virus broadly neutralizing antibody. PLoS Pathog. 2019;15(1):e1007427. Epub 20190103. doi: 10.1371/journal.ppat.1007427 ; PubMed Central PMCID: PMC6317788. 30605488 PMC6317788

[60] Tiller T, Meffre E, Yurasov S, Tsuiji M, Nussenzweig MC, Wardemann H. Efficient generation of monoclonal antibodies from single human B cells by single cell RT-PCR and expression vector cloning. J Immunol Methods. 2008;329(1–2):112–24. Epub 20071031. doi: 10.1016/j.jim.2007.09.017 ; PubMed Central PMCID: PMC2243222. 17996249 PMC2243222

[61] Kabsch W. Xds. Acta Crystallogr D Biol Crystallogr. 2010;66(Pt 2):125–32. Epub 20100122. doi: 10.1107/S0907444909047337 ; PubMed Central PMCID: PMC2815665. 20124692 PMC2815665

[62] McCoy AJ, Grosse-Kunstleve RW, Adams PD, Winn MD, Storoni LC, Read RJ. Phaser crystallographic software. J Appl Crystallogr. 2007;40(Pt 4):658–74. Epub 20070713. doi: 10.1107/S0021889807021206 ; PubMed Central PMCID: PMC2483472. 19461840 PMC2483472

[63] Terwilliger TC, Grosse-Kunstleve RW, Afonine PV, Moriarty NW, Zwart PH, Hung LW, et al. Iterative model building, structure refinement and density modification with the PHENIX AutoBuild wizard. Acta Crystallogr D Biol Crystallogr. 2008;64(Pt 1):61–9. Epub 20071205. doi: 10.1107/S090744490705024X ; PubMed Central PMCID: PMC2394820. 18094468 PMC2394820

[64] Emsley P, Cowtan K. Coot: model-building tools for molecular graphics. Acta Crystallogr D Biol Crystallogr. 2004;60(Pt 12 Pt 1):2126–32. Epub 20041126. doi: 10.1107/S0907444904019158 . 15572765

[65] Adams PD, Afonine PV, Bunkoczi G, Chen VB, Davis IW, Echols N, et al. PHENIX: a comprehensive Python-based system for macromolecular structure solution. Acta Crystallogr D Biol Crystallogr. 2010;66(Pt 2):213–21. Epub 20100122. doi: 10.1107/S0907444909052925 ; PubMed Central PMCID: PMC2815670. 20124702 PMC2815670

[66] Pettersen EF, Goddard TD, Huang CC, Couch GS, Greenblatt DM, Meng EC, et al. UCSF Chimera—a visualization system for exploratory research and analysis. J Comput Chem. 2004;25(13):1605–12. doi: 10.1002/jcc.20084 . 15264254

[67] Raveh B, London N, Schueler-Furman O. Sub-angstrom modeling of complexes between flexible peptides and globular proteins. Proteins. 2010;78(9):2029–40. doi: 10.1002/prot.22716 . 20455260

[68] Leman JK, Weitzner BD, Lewis SM, Adolf-Bryfogle J, Alam N, Alford RF, et al. Macromolecular modeling and design in Rosetta: recent methods and frameworks. Nat Methods. 2020;17(7):665–80. Epub 20200601. doi: 10.1038/s41592-020-0848-2 ; PubMed Central PMCID: PMC7603796. 32483333 PMC7603796

[69] Barlow KA, S OC, Thompson S, Suresh P, Lucas JE, Heinonen M, et al. Flex ddG: Rosetta Ensemble-Based Estimation of Changes in Protein-Protein Binding Affinity upon Mutation. J Phys Chem B. 2018;122(21):5389–99. Epub 20180215. doi: 10.1021/acs.jpcb.7b11367 ; PubMed Central PMCID: PMC5980710. 29401388 PMC5980710

[70] Alford RF, Leaver-Fay A, Jeliazkov JR, O’Meara MJ, DiMaio FP, Park H, et al. The Rosetta All-Atom Energy Function for Macromolecular Modeling and Design. J Chem Theory Comput. 2017;13(6):3031–48. Epub 20170512. doi: 10.1021/acs.jctc.7b00125 ; PubMed Central PMCID: PMC5717763. 28430426 PMC5717763

[71] Wu Y, Wang F, Shen C, Peng W, Li D, Zhao C, et al. A noncompeting pair of human neutralizing antibodies block COVID-19 virus binding to its receptor ACE2. Science. 2020;368(6496):1274–8. Epub 20200513. doi: 10.1126/science.abc2241 ; PubMed Central PMCID: PMC7223722. 32404477 PMC7223722

