# Supplementary figures and images for: Structural basis for cross-group recognition of an influenza virus hemagglutinin antibody that targets postfusion stabilized epitope
Source: PLoS Pathog. 2023 Aug 9;19(8):e1011554. doi: 10.1371/journal.ppat.1011554 (PMC10411744; doi:10.1371/journal.ppat.1011554)

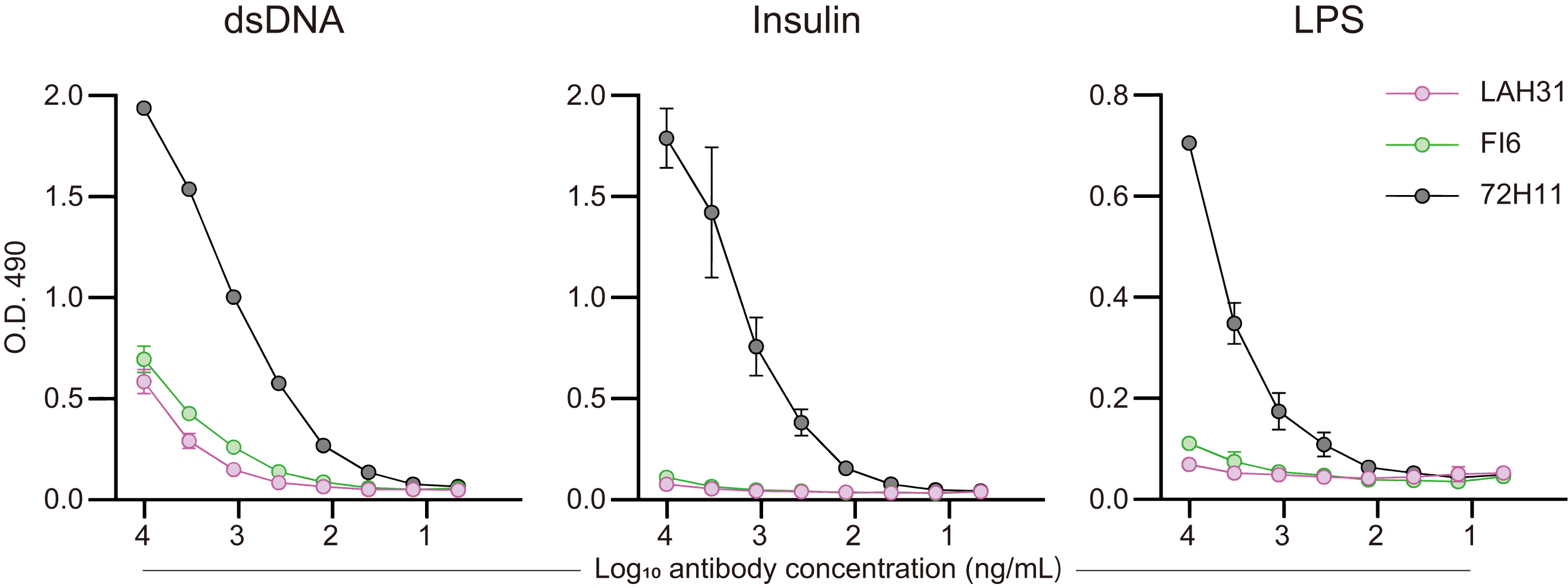

Supplement: S1 Fig — The binding ability of LAH31 against dsDNA, insulin or LPS was assessed using ELISA. FI6 and 72H11 were used for comparison and as a positive control, respectively. The Assay was conducted in duplicates and representative data from two independent experiments are represented as mean ± SD. (TIF) [file ppat.1011554.s001.tif]

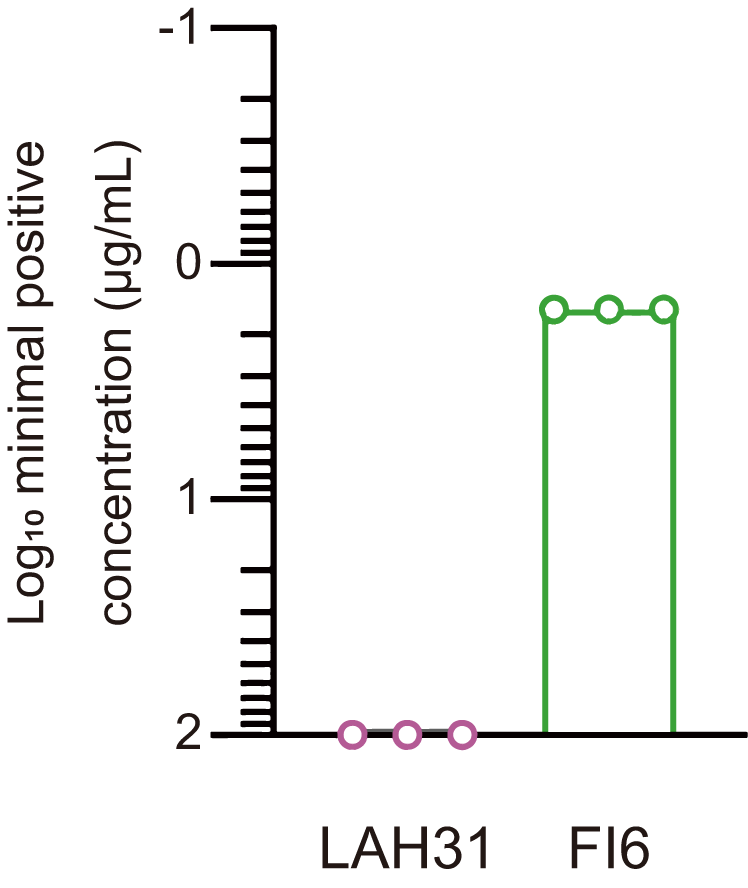

Supplement: S2 Fig — Serially diluted mAbs were applied to the virus-neutralization assay using X31 virus. FI6 was used as a positive control. Minimal neutralizing titer is shown (100 μg/ml as maximum). Bars represent the mean. (TIF) [file ppat.1011554.s002.tif]

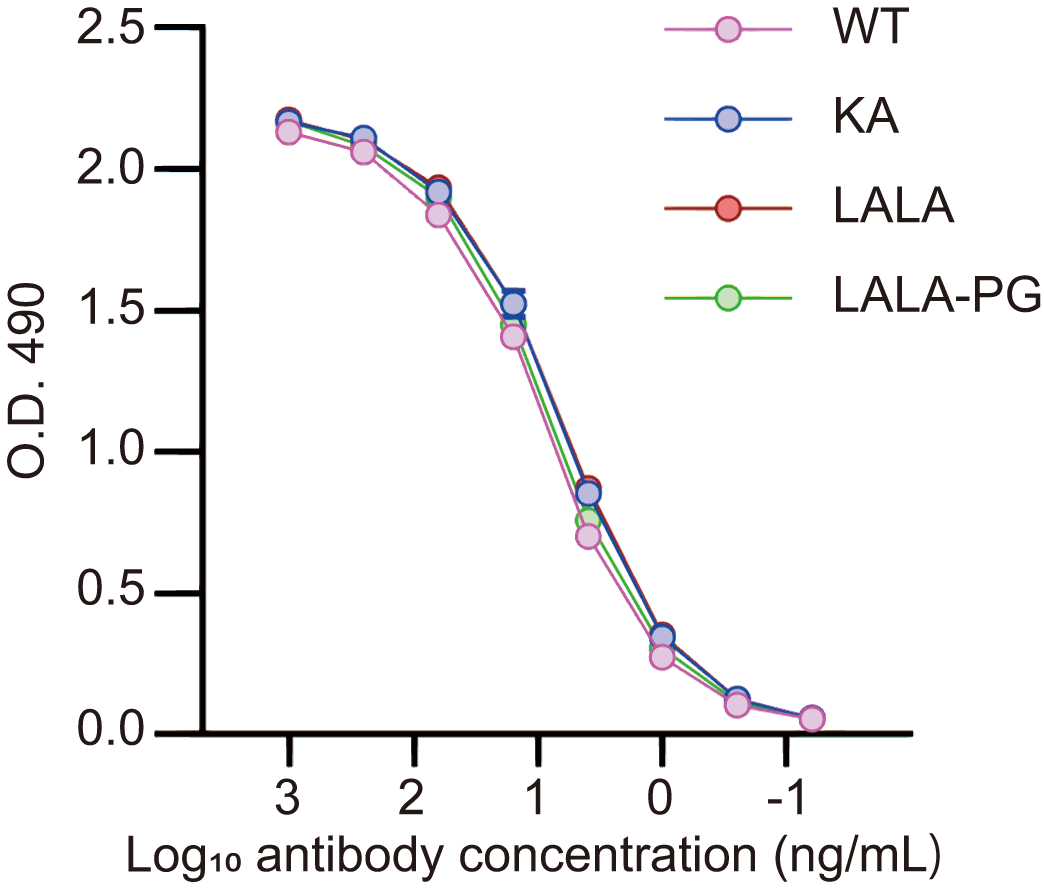

Supplement: S3 Fig — The binding ability of WT and Fc mutated (KA, LALA, LALA-PG) LAH31 against X31 HA was assessed using ELISA. The Assay was conducted in duplicates and representative data from two independent experiments are represented as mean ± SD. (TIF) [file ppat.1011554.s003.tif]

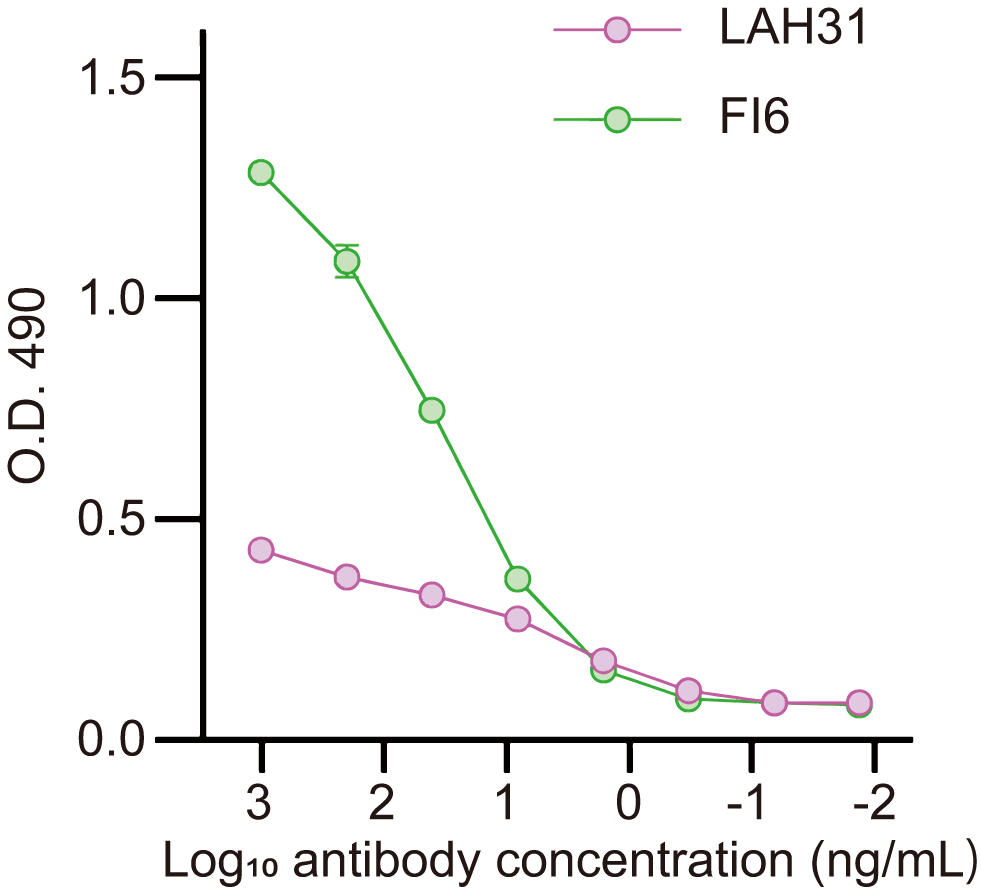

Supplement: S4 Fig — The binding ability of LAH31 against commercial influenza split vaccine (2022–2023 season) was assessed using ELISA. The vaccine antigens were captured by pre-coated S5V2-29. FI6 was used as a positive control. The Assay was conducted in triplicates and representative data from two independent experiments are represented as mean ± SD. (TIF) [file ppat.1011554.s004.tif]

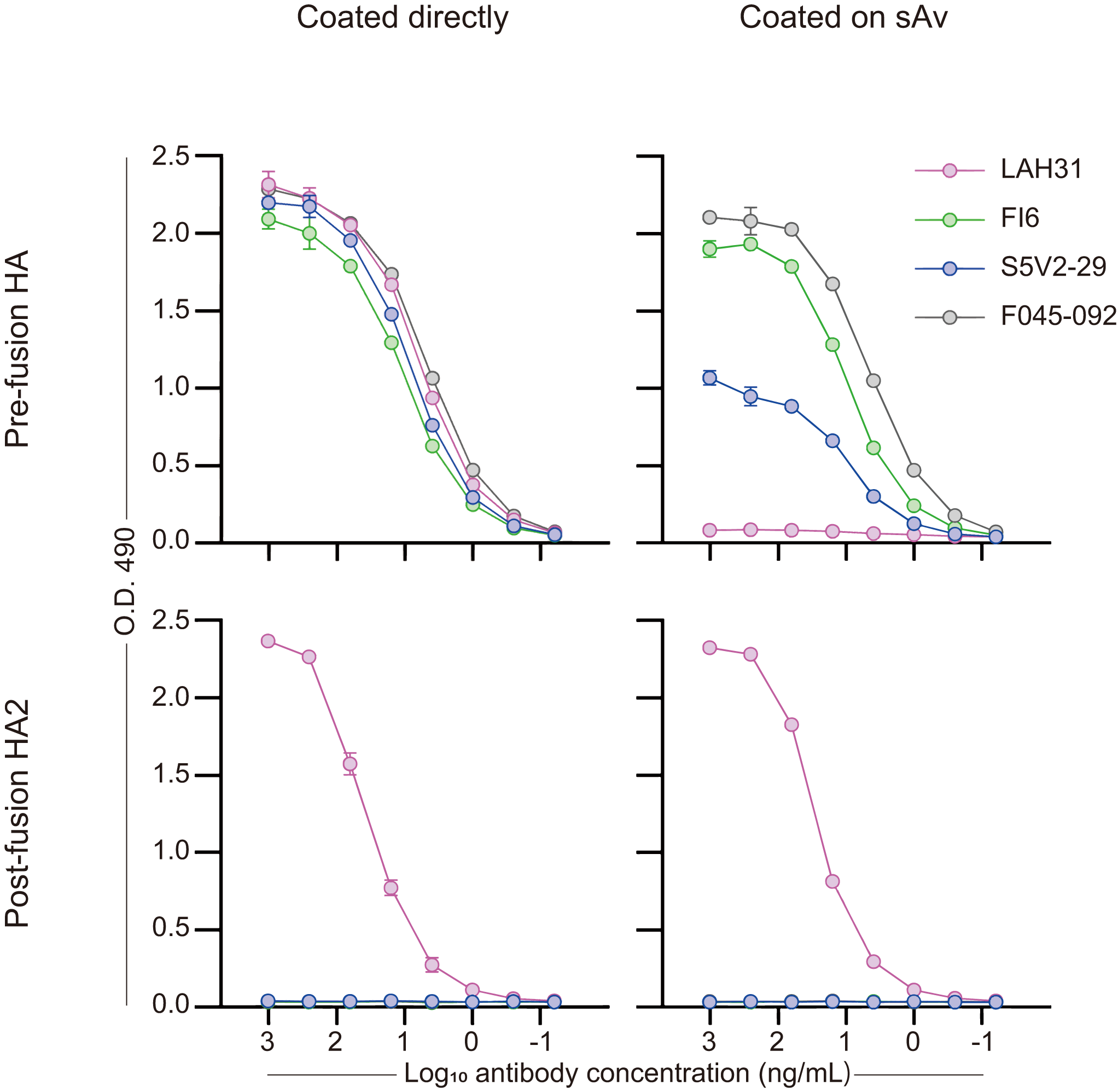

Supplement: S5 Fig — LAH31 and published mAbs with different epitope specificity (FI6, CS; S5V2-29, HI; F045-092, RBS) were tested for binding to X31 (H3N2) prefusion HA (top) or postfusion HA2 (bottom). For each antigen, ELISA was performed by coating directly (left) or via-streptavidin (right). The Assay was conducted in duplicates and representative data from two independent experiments are represented as mean ± SD. (TIF) [file ppat.1011554.s005.tif]

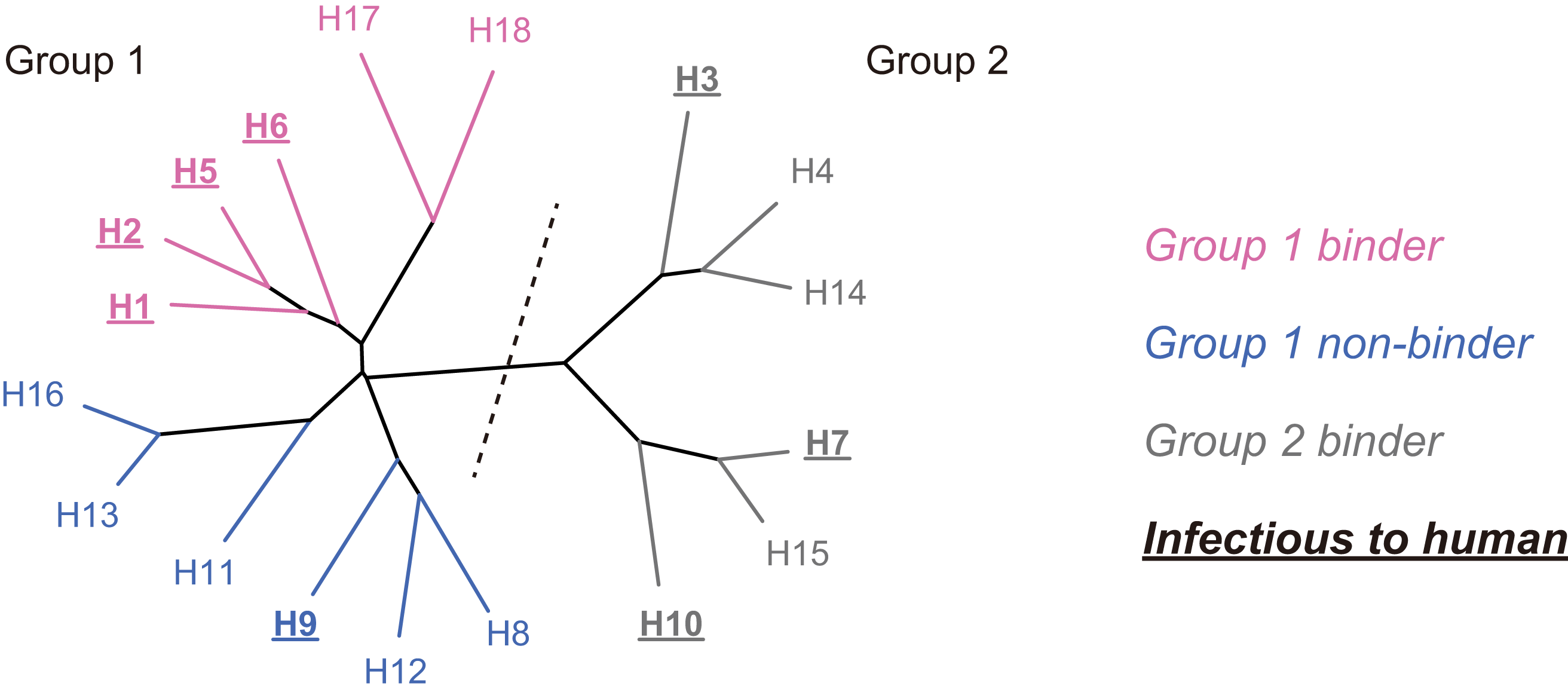

Supplement: S6 Fig — Each node is colored based on whether LAH31 binds to (pink, group 1; grey, group 2) or not (blue). Subtypes reported to be infectious to human are underlined. (TIF) [file ppat.1011554.s006.tif]

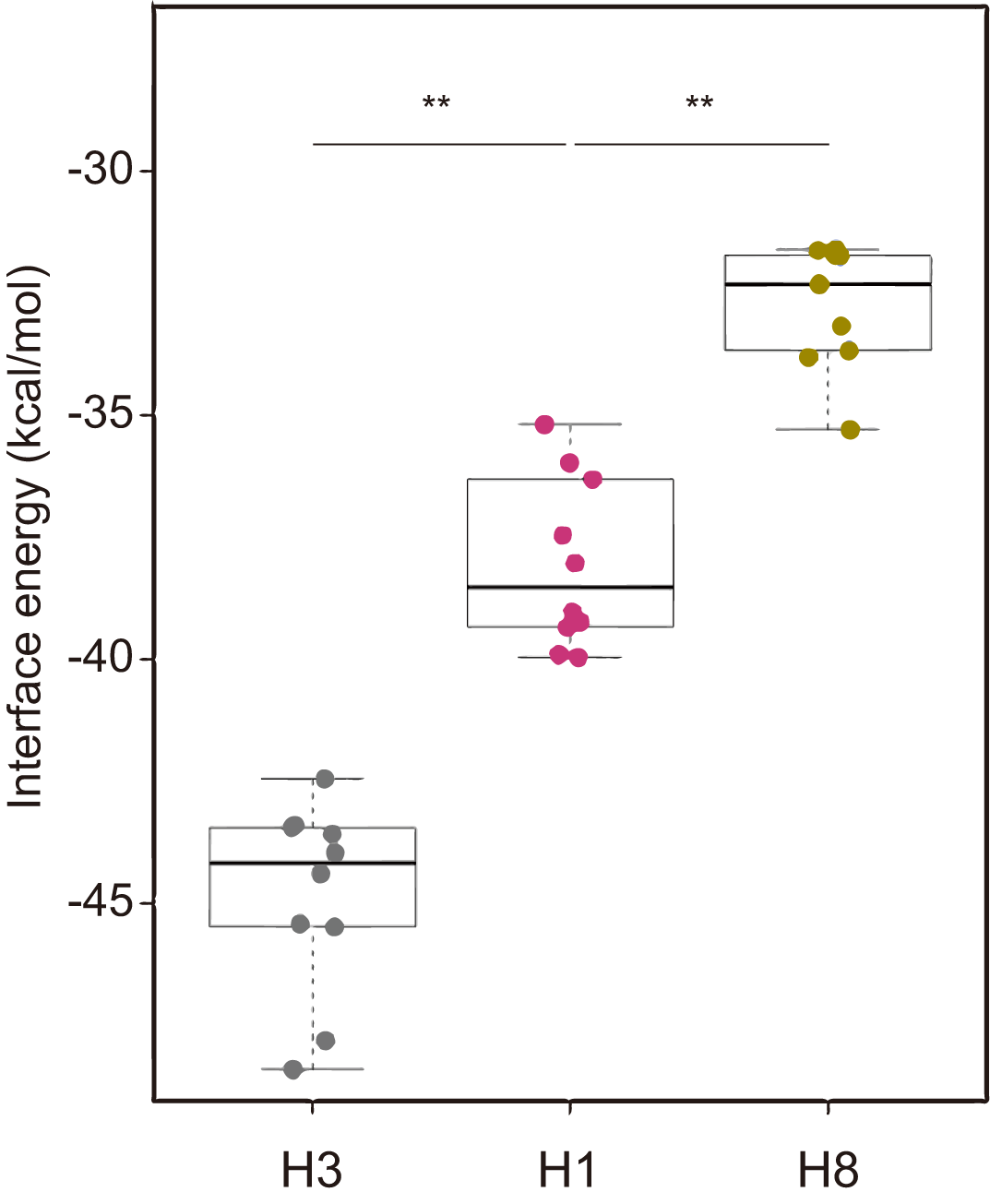

Supplement: S7 Fig — 200 model structures of complex in each subtype were produced from experimental crystal structure and the calculated energy of 10 models with highest scoring are plotted. The P values were determined with two-tailed Mann–Whitney U test. Not significant (ns), **p < 0.01. (TIF) [file ppat.1011554.s007.tif]

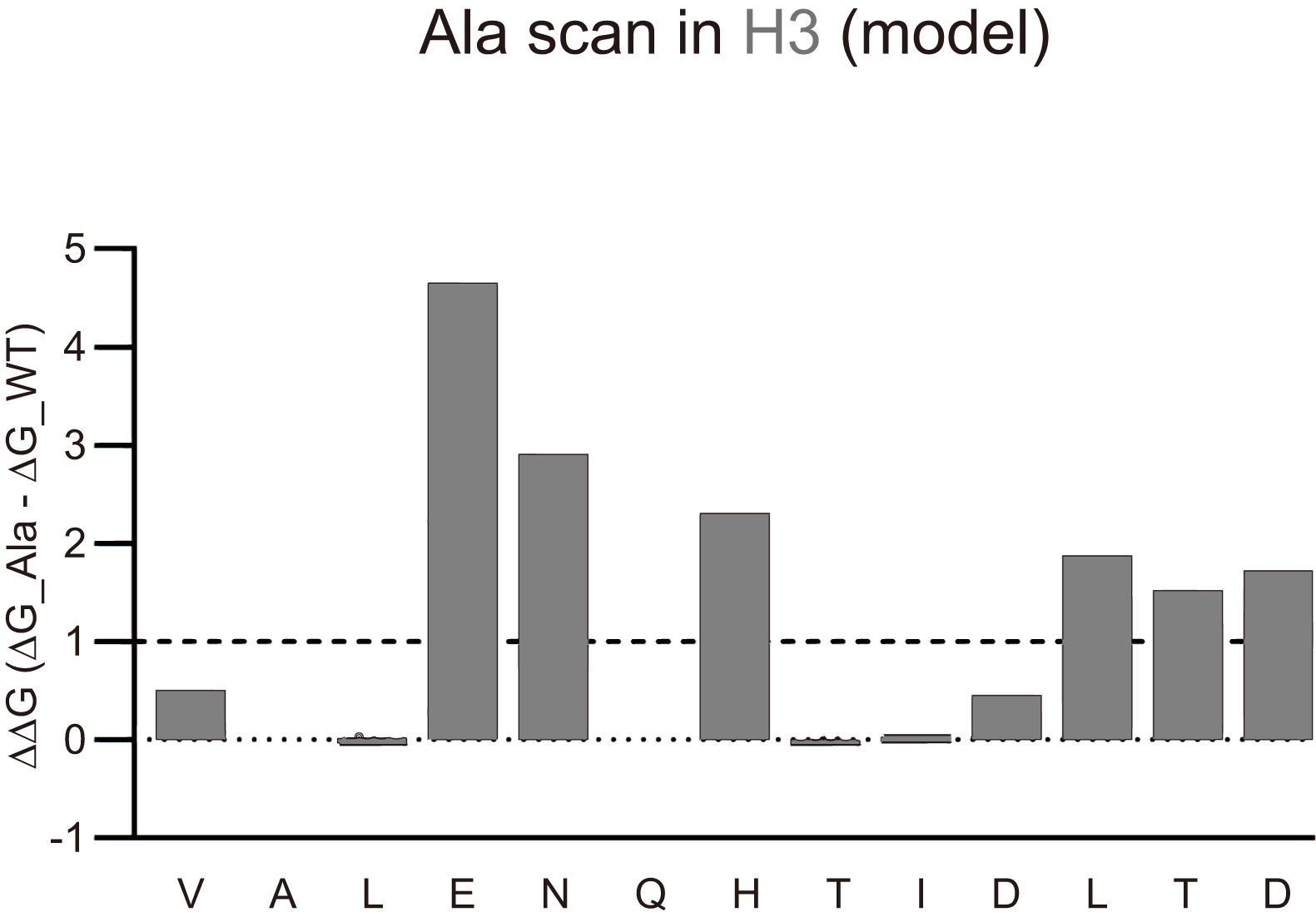

Supplement: S8 Fig — The structure prepared in S7 Fig with the highest scoring was chosen. Changes of free energy in LAH31 binding were calculated by virtually replacing corresponding residue to Ala and are indicated as a bar graph. The individual amino acid sequences are noted below each bar. (TIF) [file ppat.1011554.s008.tif]

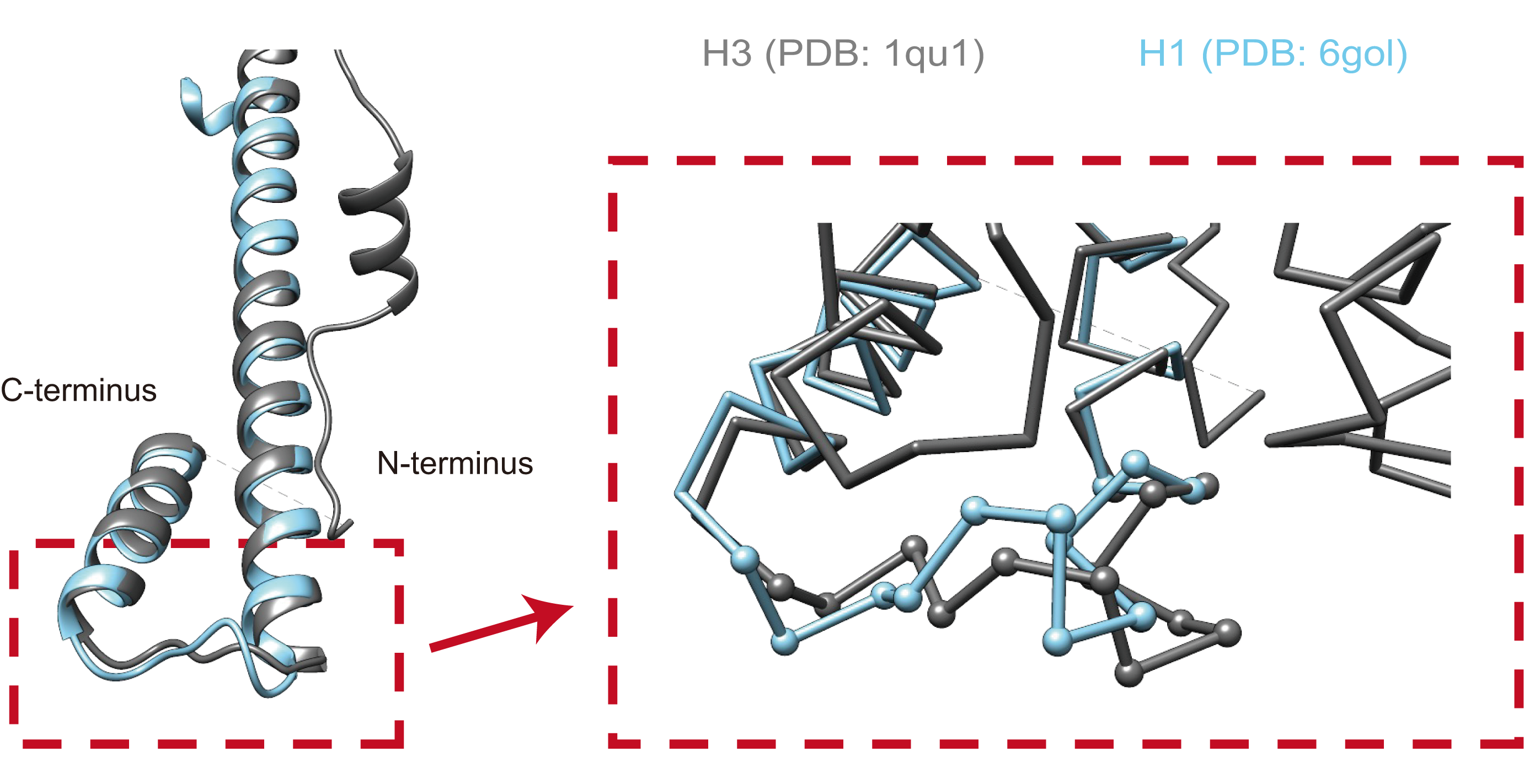

Supplement: S9 Fig — Structure of postfusion HA2 in H3 X31 (grey: 1qu1) and H1 A/Luxembourg/43/2009 (blue: 6gol) were overlayed (left). Kinked loop-helix region is further zoomed in (right). (TIF) [file ppat.1011554.s009.tif]
