# Supplementary material for: Structural basis for cross-group recognition of an influenza virus hemagglutinin antibody that targets postfusion stabilized epitope
Source: PLoS Pathog. 2023 Aug 9;19(8):e1011554. doi: 10.1371/journal.ppat.1011554 (PMC10411744; doi:10.1371/journal.ppat.1011554)
Supplement: S1 Table — (DOC) [file ppat.1011554.s010.doc]

**S1 Table. Viral strains of HA used in this study.**

| **Abbreviation** | **Strain name** | **Subtype** | **Subgroup** |
| --- | --- | --- | --- |
| PR8/34 | A/Puerto Rico/8/34 | H1N1 | Group 1 |
| NC/99 | A/New Caledonia/20/99 | H1N1 | Group 1 |
| SI/06 | A/Solomon Islands/3/2006 | H1N1 | Group 1 |
| Bris/07 | A/Brisbane/59/2007 | H1N1 | Group 1 |
| NRT/09, (p)H1* | A/Narita/1/2009 | H1N1 | Group 1 |
| Mich/15 | A/Michigan/45/2015 | H1N1 | Group 1 |
| Bris/18 | A/Brisbane/02/2018 | H1N1 | Group 1 |
| Guan/19 | A/Guangdong-Maonan/SWL1536/2019 | H1N1 | Group 1 |
| X31, H3^#^ | X31 (originated from A/Aichi/2/68) | H3N2 | Group 2 |
| Pana/99 | A/Panama/2007/99 | H3N2 | Group 2 |
| Hiro/05 | A/Hiroshima/52/2005 | H3N2 | Group 2 |
| Urg/07 | A/Uruguay/716/2007 | H3N2 | Group 2 |
| Vic/11 | A/Victoria/361/2011 | H3N2 | Group 2 |
| Sing/16 | A/Singapore/INFIMH-16-0019/2016 | H3N2 | Group 2 |
| Kans/17 | A/Kansas/14/2017 | H3N2 | Group 2 |
| Hong/19 | A/HongKong/2671/2019 | H3N2 | Group 2 |
| H1 | A/South Carolina/1/1918 | H1N1 | Group 1 |
| H2 | A/Japan/305/57 | H2N2 | Group 1 |
| H3 | A/Hong Kong/1/68 | H3N2 | Group 2 |
| H4 | A/duck/Czechoslovakia/1956 | H4N6 | Group 2 |
| H5 | A/Laos/JP127/2004 | H5N1 | Group 1 |
| H6 | A/mallard/Sweden/81/2002 | H6N1 | Group 1 |
| H7 | A/Anhui/1/2013 | H7N9 | Group 2 |
| H8 | A/mallard/Sweden/24/2002 | H8N4 | Group 1 |
| H9 | A/guinea fowl/Hong Kong/WF10/99 | H9N2 | Group 1 |
| H10 | A/mallard/Interior Alaska/10BM01929R0/2010 | H10N7 | Group 2 |
| H11 | A/northern shoveler/Netherlands/18/1999 | H11N9 | Group 1 |
| H12 | A/mallard/Interior Alaska/7MP0167/2007 | H12N5 | Group 1 |
| H13 | A/black-headed gull/Sweden/1/1999 | H13N6 | Group 1 |
| H14 | A/mallard/Astrakhan/263/1982 | H14N5 | Group 2 |
| H15 | A/shearwater/Australia/2576/1979 | H15N9 | Group 2 |
| H16 | A/black-headed gull/Sweden/5/99 | H16N3 | Group 1 |
| H17 | A/little yellow-shouldered bat/Guatemala/060/2010 | H17N10 | Group 1 |
| H18 | A/flat-faced bat/Peru/033/2010 | H18N11 | Group 1 |

*H1 in Fig 5C stands for A/Narita/1/2009.

^#^H3 in Fig 5C stands for X31.
