# Supplementary material for: Structural basis for cross-group recognition of an influenza virus hemagglutinin antibody that targets postfusion stabilized epitope
Source: PLoS Pathog. 2023 Aug 9;19(8):e1011554. doi: 10.1371/journal.ppat.1011554 (PMC10411744; doi:10.1371/journal.ppat.1011554)
Supplement: S2 Table — (DOC) [file ppat.1011554.s011.doc]

**S2 Table. Data collection and refinement statistics.**

|  | LAH31-LAH peptide  Complex |
| --- | --- |
| **Data collection** |  |
| Space group | *P*2_1_2_1_2 |
| Cell dimensions |  |
| *a*, *b*, *c* (Å) | 68.05, 87.51, 89.97 |
| Resolution (Å) | 45.98-1.95 (2.06-1.95)* |
| *R*_meas_ (%) | 21.5 (92.7) |
| CC_1/2_ (%) | 99.4 (65.4) |
| *<I* / σ(*I)>* | 10.0 (1.93) |
| Completeness (%) | 98.7 (92.3) |
| Redundancy | 6.2 (4.1) |
| **Refinement** |  |
| No. reflections | 39125 |
| *R*_work_ / *R*_free_ (%) | 18.5/22.5 |
| No. atoms |  |
| Protein | 3271 |
| Ligand | 102 |
| Water | 304 |
| *B*-factors (Å^2^) |  |
| Protein | 32.5 |
| Ligand | 36.0 |
| Water | 38.1 |
| R.m.s. deviations |  |
| Bond lengths (Å) | 0.005 |
| Bond angles (°) | 0.763 |
| Ramchandran Statistics (%) |  |
| Favored | 98.18 |
| Outliers | 0.46 |

*Values in parentheses are for highest-resolution shell.
