# Supplementary material for: Structural basis for cross-group recognition of an influenza virus hemagglutinin antibody that targets postfusion stabilized epitope
Source: PLoS Pathog. 2023 Aug 9;19(8):e1011554. doi: 10.1371/journal.ppat.1011554 (PMC10411744; doi:10.1371/journal.ppat.1011554)
Supplement: S3 Table — The residue number and amino acid sequence of HA2 region of H3 X31 HA used in this study is shown. Residues are numbered in H3 numbering. LAH region is highlighted by bold capital and underline in the sequence. (DOC) [file ppat.1011554.s012.doc]

**S3 Table.** **Sequence of HA2 region used for postfusion HA2 antigen in this study.**

| **Residue #** | **Amino acid sequence** |
| --- | --- |
| 24 - 185 | FRHQNSEGTG QAADLKSTQA AIDQINGKLN RVIEKTNEKF HQIEKEFSEV EG**RIQDLEKY VEDTKIDLWS YNAELLVALE NQHTIDLTDS EMNKLFEKTR RQLRENA**EDM GNGSFKIYHK CDNACIESIR NGTYDHDVYR DEALNNRFQI KGVELKSGYK DW |
