# Supplementary material for: Structural basis for cross-group recognition of an influenza virus hemagglutinin antibody that targets postfusion stabilized epitope
Source: PLoS Pathog. 2023 Aug 9;19(8):e1011554. doi: 10.1371/journal.ppat.1011554 (PMC10411744; doi:10.1371/journal.ppat.1011554)
Supplement: S4 Table — (DOC) [file ppat.1011554.s013.doc]

**S4 Table. Contact residues located within 4Å distance between LAH31 and epitope LAH peptide.**

| **LAH peptide residue** | | **LAH31 residue** | | **LAH31 chain** | **LAH31 region** | **Specific interaction** |
| --- | --- | --- | --- | --- | --- | --- |
| 103 | GLU | 94 | SER | Light | CDR-K3 | Hydrogen bond |
| 104 | ASN | 92 | ARG | Light | CDR-K3 |  |
| 104 | ASN | 93 | GLN | Light | CDR-K3 | Hydrogen bond |
| 104 | ASN | 94 | SER | Light | CDR-K3 | Hydrogen bond |
| 104 | ASN | 95 | SER | Light | CDR-K3 | Hydrogen bond |
| 105 | GLN | 27d | GLN | Light | CDR-K1 | Hydrogen bond |
| 106 | HIS | 28 | ASN | Light | CDR-K1 |  |
| 106 | HIS | 32 | TYR | Light | CDR-K1 | π-π |
| 106 | HIS | 91 | ALA | Light | CDR-K3 | Hydrogen bond |
| 106 | HIS | 92 | ARG | Light | CDR-K3 |  |
| 106 | HIS | 94 | SER | Light | CDR-K3 |  |
| 103 | GLU | 50 | ARG | Heavy | FR-H2 | Salt bridge |
| 103 | GLU | 52 | ILE | Heavy | CDR-H2 |  |
| 103 | GLU | 97 | SER | Heavy | CDR-H3 | Hydrogen bond |
| 103 | GLU | 98 | GLY | Heavy | CDR-H3 | Hydrogen bond |
| 106 | HIS | 98 | GLY | Heavy | CDR-H3 |  |
| 106 | HIS | 99 | SER | Heavy | CDR-H3 |  |
| 106 | HIS | 100a | TYR | Heavy | CDR-H3 |  |
| 107 | THR | 99 | SER | Heavy | CDR-H3 |  |
| 108 | ILE | 97 | SER | Heavy | CDR-H3 |  |
| 108 | ILE | 98 | GLY | Heavy | CDR-H3 |  |
| 108 | ILE | 99 | SER | Heavy | CDR-H3 | Hydrogen bond |
| 109 | ASP | 97 | SER | Heavy | CDR-H3 | Hydrogen bond |
| 109 | ASP | 99 | SER | Heavy | CDR-H3 |  |
| 109 | ASP | 100 | SER | Heavy | CDR-H3 | Hydrogen bond |
| 110 | LEU | 54 | VAL | Heavy | CDR-H2 |  |
| 110 | LEU | 56 | LEU | Heavy | CDR-H2 |  |
| 110 | LEU | 97 | SER | Heavy | CDR-H3 | Hydrogen bond |
| 111 | THR | 30 | SER | Heavy | CDR-H1 |  |
| 111 | THR | 31 | ARG | Heavy | CDR-H1 |  |
| 111 | THR | 32 | SER | Heavy | CDR-H1 |  |
| 111 | THR | 52 | ILE | Heavy | CDR-H2 |  |
